# Development of an optogenetics tool, Opto-RANK, for control of osteoclast differentiation using blue light

**DOI:** 10.1038/s41598-024-52056-w

**Published:** 2024-01-19

**Authors:** Aiko Takada, Toshifumi Asano, Ken-ichi Nakahama, Takashi Ono, Takao Nakata, Tomohiro Ishii

**Affiliations:** 1 Department of Orthodontic Science, Graduate School of Medical and Dental Science, Tokyo Medical and Dental University (TMDU), Tokyo, 113-8510 Japan; 2 Department of Cell Biology, Graduate School of Medical and Dental Science, Tokyo Medical and Dental University (TMDU), Tokyo, 113-8510 Japan; 3 Department of Cellular Physiological Chemistry, Graduate School of Medical and Dental Science, Tokyo Medical and Dental University (TMDU), Tokyo, 113-8510 Japan; 4 The Center for Brain Integration Research (CBIR), Tokyo Medical and Dental University (TMDU), Tokyo, 113-8510 Japan; 5 Present Address: Center for Integrative Biosciences, Tokyo Institute of Technology, Yokohama, 226-8501 Japan

**Keywords:** Biological techniques, Cell biology, Chemical biology, Developmental biology

## Abstract

Optogenetics enables precise regulation of intracellular signaling in target cells. However, the application of optogenetics to induce the differentiation of precursor cells and generate mature cells with specific functions has not yet been fully explored. Here, we focused on osteoclasts, which play an important role in bone remodeling, to develop a novel optogenetics tool, Opto-RANK, which can manipulate intracellular signals involved in osteoclast differentiation and maturation using blue light. We engineered Opto-RANK variants, Opto-RANKc and Opto-RANKm, and generated stable cell lines through retroviral transduction. Differentiation was induced by blue light, and various assays were conducted for functional analysis. Osteoclast precursor cells expressing Opto-RANK differentiated into multinucleated giant cells on light exposure and displayed upregulation of genes normally induced in differentiated osteoclasts. Furthermore, the differentiated cells exhibited bone-resorbing activities, with the possibility of spatial control of the resorption by targeted light illumination. These results suggested that Opto-RANK cells differentiated by light possess the features of osteoclasts, both morphological and functional. Thus, Opto-RANK should be useful for detailed spatiotemporal analysis of intracellular signaling during osteoclast differentiation and the development of new therapies for various bone diseases.

## Introduction

Optogenetics allows for precise, non-invasive, and reversible spatiotemporal control of target molecules using light. Over the last decade, protein engineering technologies have enabled the extension of optogenetic applications to control various protein functions and cellular events outside of the nervous system^1,2^. *Arabidopsis* Cryptochrome 2 (CRY2) is a blue light-responsive photosensor that binds cryptochrome-interacting basic-helix-loop-helix 1 (CIB1) in its photoexcited state^3^. In addition, CRY2 homo-oligomerizes upon exposure to blue light^4^. Photosensory modules such as CRY2 and light-oxygen-voltage sensing domain 2 (LOV2) have been fused to target proteins to enable light-induced protein self-oligomerization, protein-target heterodimerization, protein-target dissociation, and optical allosteric control of protein activity^1,5–7^. Especially in cell differentiation studies, optogenetic tools for extracellular signal-regulated kinase (ERK), Son of sevenless homolog-2 (SOS2), and tropomyosin receptor kinase B have been developed to induce neurite outgrowth and differentiation of PC12 cells^1,6,8,9^. In addition, optogenetic control of the transcription factor Achaete-scute homolog 1 in neuronal precursors revealed the mechanisms of fate choice^10^. Although various optogenetic tools have been developed, the generation of mature differentiated cells with specific functions from their precursors using only optogenetic tools has not yet been fully explored.

Bone plays an essential role in locomotion, body protection, hematopoiesis, and mineral homeostasis^11–13^. It is a dynamic organ that constantly undergoes resorption by osteoclasts and bone formation by osteoblasts. The cooperative activity of osteoclasts and osteoblasts maintains proper bone shape, volume, and density. Osteoclasts are large multinucleated cells that secrete acids and proteases into the resorptive zone to degrade the mineral and organic phases of bone, respectively. Osteoclasts are differentiated from cells of the monocyte/macrophage lineage by macrophage colony-stimulating factor (M-CSF) and receptor activator of nuclear factor-kB ligand (RANKL). M-CSF promotes the proliferation of these osteoclast precursor cells and induces the expression of receptor activator of nuclear factor-kB (RANK)^14^, a transmembrane protein expressed in the osteoclastic lineage and the receptor for RANKL^15–17^. RANKL activates RANK, as well as subsequent downstream signaling pathways, including nuclear factor-kB and mitogen-activated protein kinase (MAPK) signaling, via tumor necrosis factor receptor-associated factor 6 (TRAF6)^11,12,18,19^. When co-stimulated via receptors such as osteoclast-associated Ig-like receptor (OSCAR) and triggering receptor expressed on myeloid cells 2 (TREM2), which associate with FcRγ or Dap12, adaptor proteins that possess an immunoreceptor tyrosine-based activation motif, the RANK signaling pathway activates nuclear factor of activated T cells, cytoplasmic 1 (Nfatc1), a master transcription factor in osteoclastogenesis^11–13,20,21^. RANKL function is inhibited by the decoy receptor osteoprotegerin (OPG), such that osteoclast precursor cells cannot be differentiated in the presence of OPG^22,23^. In bone biology, an optogenetic tool for Plexin-B1 was developed to study contact repulsion between osteoclasts and osteoblasts^24^. Subcellular activation of Plexin at the leading edge of migrating osteoblasts by light induces local retraction and distal protrusions to steer cells away. Differentiation of MC3T3-E1 cells, a model cell line for pre-osteoblast differentiation to osteoblasts, is promoted by increased intracellular Ca^2+^ signaling induced by the Ca^2+^-controllable optogenetic tool BACCS^25,26^.

RAW264.7 cells are a monocyte/macrophage-like cell lineage derived from Balb/c mice infected with Abelson leukemia virus^27,28^. These respond to RANKL and are subsequently differentiated into multinucleated cells with the characteristics of mature differentiated osteoclasts^29^. RAW264.7 cells have been used to study osteoclastogenesis for over 20 years and are, therefore, an accepted cell model for osteoclastogenesis studies^27^. A cell engineering approach has been developed to control the differentiation of RAW264.7 cells into osteoclasts^30^. This system is based on chemical dimerization inducer technology, which uses drugs to control RANK activation, thereby inducing RAW264.7 cells to differentiate into osteoclasts. While such a chemical-based approach is a reliable method to generate osteoclasts in a time-controlled manner, common limitations associated with chemical-based approaches include potential side effects, difficulty in removing inducers, low temporal accuracy due to extended diffusion time, and poor spatial accuracy.

We aimed to develop Opto-RANK, a spatially controllable optogenetic tool designed for the activation of the RANK signaling cascades in RAW264.7 cells, to induce them to differentiate into mature osteoclasts with the ability to form resorptive lacunae and pits on calcium phosphate (CaP)-coated surface.

## Results

### Engineering of optogenetic tools for RANK activation

**Figure 1:**
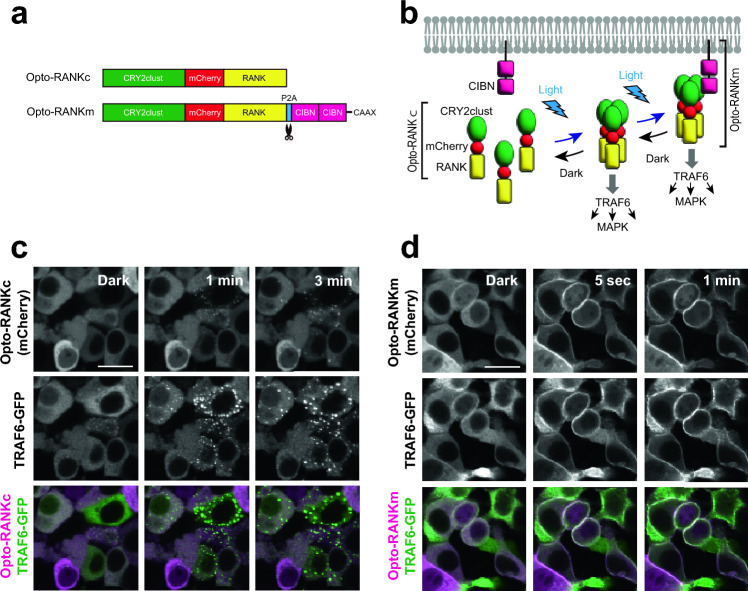
Schematic representation and in vitro optical activation of Opto-RANK. (**a**) Schematic representation of the two Opto-RANK constructs, Opto-RANKc and Opto-RANKm. The cytoplasmic domain of the RANK transmembrane protein was used in the constructs. Opto-RANKc consists of the photosensory module CRY2clust, mCherry, and the cytoplasmic domain of RANK. Opto-RANKm contains the self-cleaving P2A peptide, tandem CIBN, and the CAAX motif (at the C-terminus), in addition to the complete Opto-RANKc. (**b**) Schematic design of RANK activation using Opto-RANK. Opto-RANKc, consisting of CRY2clust, mCherry, and the cytoplasmic domain of RANK, is distributed throughout the cytosol. Upon blue light exposure, Opto-RANKc forms clusters, resulting in the binding of TRAF6 to the oligomerized RANK domain and subsequent activation of downstream signaling cascades. In the case of Opto-RANKm, light-activated Opto-RANKc is recruited to the membrane-anchored CIBN, resulting in membrane localization of the oligomerized RANK. (**c**) Representative images of HEK293T cells expressing Opto-RANKc and GFP-TRAF6. The cells were illuminated with a 488 nm wavelength laser every 5 s. Images of Opto-RANKc, TRAF6-GFP, and their merge before illumination (dark) and 1 and 3 min after the first illumination. Scale bar: 20 μm. (**d**) Representative images of HEK293T cells expressing Opto-RANKm and GFP-TRAF6. The cells were illuminated with a 488 nm wavelength laser every 5 s. Images of Opto-RANKc, TRAF6-GFP, and their merge, before illumination (dark) and 5 s and 1 min after the first illumination. Scale bar: 20 μm. Ratio of GFP-TRAF6 positive cells among Opto-RANK positive cells: 95.2% (139 out of 146 cells) in (**c**) and 94.8% (127 out of 134 cells) in (**d**) from three independent experiments. In the images, all Opto-RANK positive cells co-express GFP-TRAF6. RANK, receptor activator of nuclear factor-kB; TRAF6, tumor necrosis factor receptor-associated factor 6; GFP, green fluorescent protein.

We engineered two optogenetic tools, Opto-RANKc (c: cytoplasmic) and Opto-RANKm (m: membrane), recognizing that the RANK signaling pathway is activated by clustering the RANK cytoplasmic signaling domain^30^. To control light-induced homo-oligomerization of the RANK cytoplasmic domain, we fused CRY2clust, which enables robust clustering of CRY2 upon blue light illumination^31^, together with the red fluorescent protein mCherry, to the RANK domain (Fig. 1a and Supplementary Fig. S1). This optogenetic fusion protein Opto-RANKc clusters in response to blue light, leading to the activation of the RANK downstream signaling cascades (Fig. 1b). We also generated a construct with the light-induced membrane-recruiting ability of Opto-RANKc, termed Opto-RANKm (Fig. 1a and Supplementary Fig. S1). In the Opto-RANKm construct, Opto-RANKc was linked to the self-cleaving porcine teschovirus-1 2A (P2A) peptide, the tandem repeat of CIBN (N-terminus of *Arabidopsis thaliana* CIB1), which is a heterodimerization partner of photoactivated CRY2, and the CAAX motif of K-Ras^32^. In this case, blue light induces oligomerization of Opto-RANKc and simultaneously recruits it to the membrane through direct interaction with the double CIBN tethered to the membrane via the CAAX motif (Fig. 1b).

To observe the response of Opto-RANK to blue light in vitro, *Opto-RANK* and *GFP-TRAF6* were co-transfected into HEK293T cells. TRAF6 is a critical RANK-binding signaling molecule that regulates osteoclast differentiation^18,19^. Live imaging of cells expressing Opto-RANKc showed that Opto-RANKc formed aggregates in response to blue light, whereas GFP-TRAF6 exhibited robust aggregation (Fig. 1c). Cells expressing Opto-RANKm were also observed, and both Opto-RANKm and GFP-TRAF6 readily translocated to the membrane within 5 s of exposure to blue light (Fig. 1d). The observation that Opto-RANK responds to blue light and recruits TRAF6 suggests that these optogenetic tools can activate TRAF6 upon blue light irradiation and potentially activate downstream intracellular signaling cascades that control subsequent osteoclast differentiation.

### Evaluation of the differentiation potential of Opto-RANK-expressing RAW264.7 cells into osteoclasts

**Figure 2:**
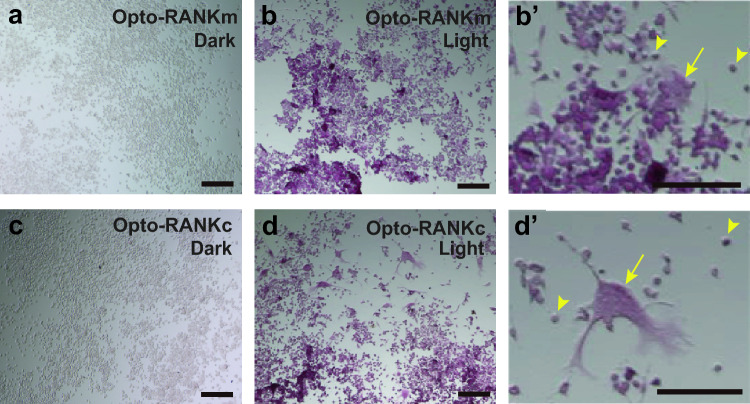
Analysis of the responsiveness of Opto-RANK cells to blue light using TRAP staining. (**a**–**b**) Opto-RANKm cells were cultured on a collagen-coated plate without (**a**) or with (**b**) blue light exposure every 2 min, for 5 d, and then stained for TRAP. (**b**′) A higher magnification view of the staining in (**b**). (**c**–**d**) Opto-RANKc cells were cultured on a collagen-coated plate without (**c**) or with (**d**) blue light exposure every 2 min for 5 d and then stained for TRAP. (**d**′) Higher magnification views of the staining in (**d**). Arrows and arrow heads indicate multinucleated and mononuclear cells, respectively. Scale bar: 150 μm. RANK, receptor activator of nuclear factor-kB; TRAP, tartrate-resistant acid phosphatase.

To investigate whether Opto-RANK can induce osteoclast precursor cell differentiation by blue light, we evaluated the differentiation potential of cells expressing Opto-RANK. To this end, we first established clonally stable cell lines expressing these optogenetic tools. RAW264.7 cells^33^, a murine monocyte-macrophage-like cell line, were transduced with recombinant Opto-RANK retroviruses, followed by single cell cloning, resulting in the establishment of several RAW264.7 cell lines expressing Opto-RANK. The expression of Opto-RANKc and Opto-RANKm proteins was confirmed using western blot analysis (Supplementary Fig. S2). To examine the differentiation state of RAW264.7 cells, we irradiated Opto-RANK cells with blue light every 2 min for 5 d while they were cultured in a CO_2_ incubator. Subsequently, the cells were stained for TRAP, an osteoclast-associated enzyme commonly used as a sensitive indicator of osteoclast differentiation (Fig. 2). In contrast to cells cultured in the dark (Fig. 2a,c), light-illuminated cells expressing Opto-RANKc or Opto-RANKm were stained red, indicating that they differentiated into osteoclasts (Fig. 2b,d). Close-up observation of these cells revealed larger multinucleated cells in Opto-RANKc than in Opto-RANKm (Fig. 2b′,d′). We also observed multinucleated cells differentiated from different Opto-RANKc lines (Supplementary Fig. S3) and selected one of the Opto-RANKc cell lines that were efficiently differentiated by blue light for further analysis.

### Phosphorylation of MAPKs in the light-activated Opto-RANKc cells

**Figure 3:**
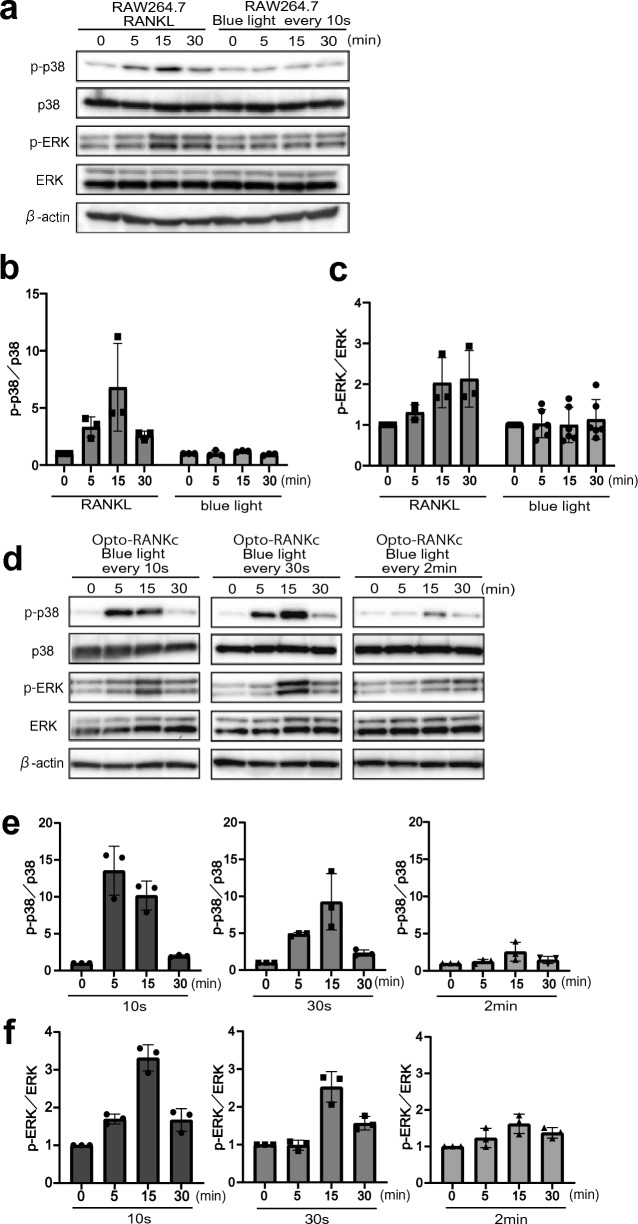
MAPK phosphorylation in RAW264.7 and Opto-RANKc cells. Phosphorylation of p38 and ERK was examined using western blot, with anti-p38, -p-p38, -ERK, and -p-ERK antibodies. β-actin was used as the loading control. (**a**) RAW264.7 cells were exposed to RANKL (left) or blue light (right) every 10 s, for 0, 5, 15, and 30 min. Representative western blot images from three independent experiments are shown. (**b**-**c**) Quantitative analysis of the data in (**a**). The band intensities for p-p38 (**b**) and p-ERK (**c**) have been normalized to those for p38 and ERK, respectively, followed by normalization to the corresponding values for 0-min RANKL/light exposure. (**d**) Opto-RANKc cells were exposed to blue light every 10 s (left), 30 s (middle), and 2 min (right) for 0, 5, 15, and 30 min. Representative western blot images from three independent experiments are shown. (**e**–**f**) Quantitative analysis of the data in (**d**). The band intensities for p-p38 (**e**) and p-ERK (**f**) have been normalized to those for p38 and ERK, respectively, followed by normalization to the corresponding values for 0-min light exposure. Cropped images of the blots are shown in (**a**) and (**d**), while the uncropped original blots are presented in Supplementary Fig. S4 online. Data in (**b**), (**c**), (**e**), and (**f**) represent the mean ± SD from three independent experiments. MAPK, mitogen-activated protein kinase; ERK, extracellular signal-regulated kinase; p-ERK, phosphorylated ERK; RANKL, receptor activator of nuclear factor-kB ligand; RANK, receptor activator of nuclear factor-kB; p-p38, phosphorylated p38.

Initial activation of RANK leads to immediate phosphorylation of MAPKs via signaling cascades, including TRAF6 and Transforming growth factor β-activated kinase 1 (TAK1). Phosphorylation of two MAPKs, p38 and ERK, was examined using western blot (Fig. 3). We first confirmed that in RAW264.7 cells, p38 and ERK were phosphorylated in the presence of RANKL, with peak phosphorylation of p38 at 15 min and elevated phosphorylation of ERK at 15 and 30 min. Moreover, there was no phosphorylation upon illumination of the cells by blue light in the absence of RANKL (Fig. 3a–c). Next, we exposed Opto-RANKc cells to blue light under three different irradiation conditions: every 10 s, 30 s, and 2 min (Fig. 3d–f). In all cases, both p38 and ERK were phosphorylated with different peak times and efficiencies: p38 was highly phosphorylated at 5 min and 15 min upon the 10-s interval stimulation and at 15 min upon the 30-s and 2-min interval stimulation (Fig. 3e). At 10-s and 30-s interval stimulations, ERK was highly phosphorylated at 15 min; however, at the 2-min interval stimulation, elevated phosphorylation was observed at 15 min and 30 min (Fig. 3f). Both p38 and ERK were most efficiently phosphorylated upon the 10-s interval stimulation. These results suggested that the immediate intracellular response of MAPK can be controlled by adjusting the frequency of optogenetic activation using Opto-RANKc.

### Differentiation of Opto-RANKc cells

**Figure 4:**
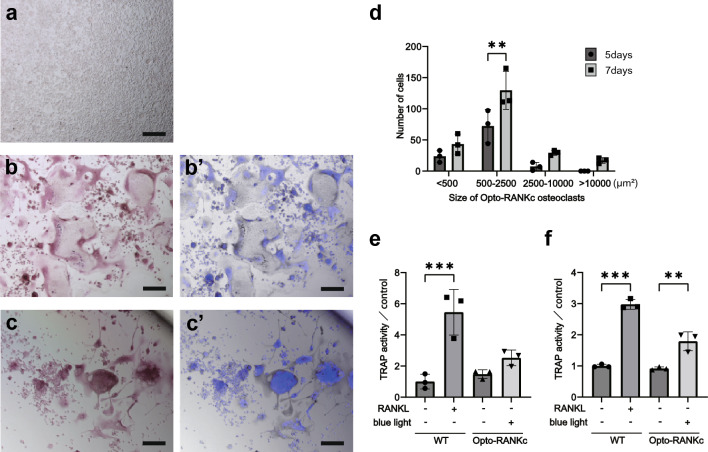
Differentiation of Opto-RANKc cells in prolonged culture. (**a**) RAW264.7 cells were cultured in RANKL-free medium for 5 d and then stained for TRAP. No TRAP-positive cells were observed. (**b**) RAW264.7 cells were cultured in RANKL-containing medium for 5 d and then stained for TRAP. (**b**′) Double staining for TRAP and DAPI in (**b**). Typical TRAP-positive multinucleated cells were observed. (**c**) Opto-RANKc cells were cultured on a collagen-coated plate with blue light illumination every 2 min for 7 d; the cells were passaged after 4 d of culture. The cells were then stained for TRAP. (**c**′) Double staining for TRAP and DAPI in (**c**). Large TRAP-positive multinucleated cells were observed. (**d**) The number of 5-d and 7-d differentiated osteoclasts of Opto-RANKc observed by TRAP staining was counted by size. (**e**–**f**) Quantitative analysis of TRAP activity. TRAP solution assays were performed. RAW264.7 (WT) and Opto-RANKc cells were differentiated with RANKL and light, respectively, for 5 d (**e**) and 7 d (**f**). Data in (**d**), (**e**), and (**f**) represent the mean ± SD from three independent experiments. Data were analyzed using one-way analysis of variance followed by Tukey’s multiple comparisons test. **, *p* < 0.01; ***, *p* < 0.001. Scale bar in (**a**)–(**c**): 150 μm. RANK, receptor activator of nuclear factor-kB; RANKL, receptor activator of nuclear factor-kB ligand; TRAP, tartrate-resistant acid phosphatase; WT, wild-type; DAPI, 4', 6-diamidino-2-phenylindole.

As mature osteoclasts are large multinucleated cells, we examined whether Opto-RANKc cells could be differentiated into such cells. First, we examined the toxicity of blue light on RAW264.7 cells. Since three different light irradiation conditions were tested in the western blot experiments, the same conditions were applied to RAW264.7 cells during the induction of differentiation using RANKL for 5 d. Blue light irradiation with the 10-s, 30-s, and 1-min intervals caused severe to mild loss of differentiated cells, as analyzed using TRAP staining (Supplementary Fig. S5), whereas irradiation with the 2-min interval resulted in the presence of TRAP-positive large osteoclasts. Although the changes in the MAPK phosphorylation in the case of the 2-min interval light stimulation were smaller than those upon RANKL stimulation, apparent changes were observed (Fig. 3d–f); therefore, this stimulation condition was chosen for further differentiation of Opto-RANKc cells. Opto-RANKc cells were cultured on collagen-coated plates with blue light irradiation every 2 min, for 7 d, with passage after 4 d of culture. We hypothesized that prolonged culture could promote osteoclast maturation because the intracellular signaling induced by Opto-RANKc appeared to be slightly weaker under this light illumination condition, based on the results of the western blot analysis. Cells differentiated from Opto-RANKc cells were multinucleated and much larger than those cultured for 5 d (Figs. 2d and 4a–d). A significant increase in TRAP activity was observed in both the 5-d and 7-d cultures, although the increase was lower in the 7-d culture (Fig. 4e-f). Thus, Opto-RANKc cells can be differentiated into large multinucleated cells upon blue light exposure, without stimulation by RANKL, although the differentiation appeared to be slower than that of RAW264.7 cells stimulated with RANKL. We also cultured Opto-RANKc cells on plates without collagen-coating and performed the same experiments, resulting in smaller differentiated cells than those observed on collagen-coated plates (Supplementary Fig. S6). This is presumably due to the difficulty of cell detachment from non-coated plates during passage compared to that from collagen-coated plates because RAW264.7 cells attach tightly to the surface of culture dishes, resulting in cell damage. Collagen, a natural ligand for OSCAR and a key molecule in the co-stimulatory pathway during osteoclast differentiation, could promote the differentiation of the Opto-RANKc cell^34,35^.

### Gene expression in Opto-RANKc differentiated cells

**Figure 5:**
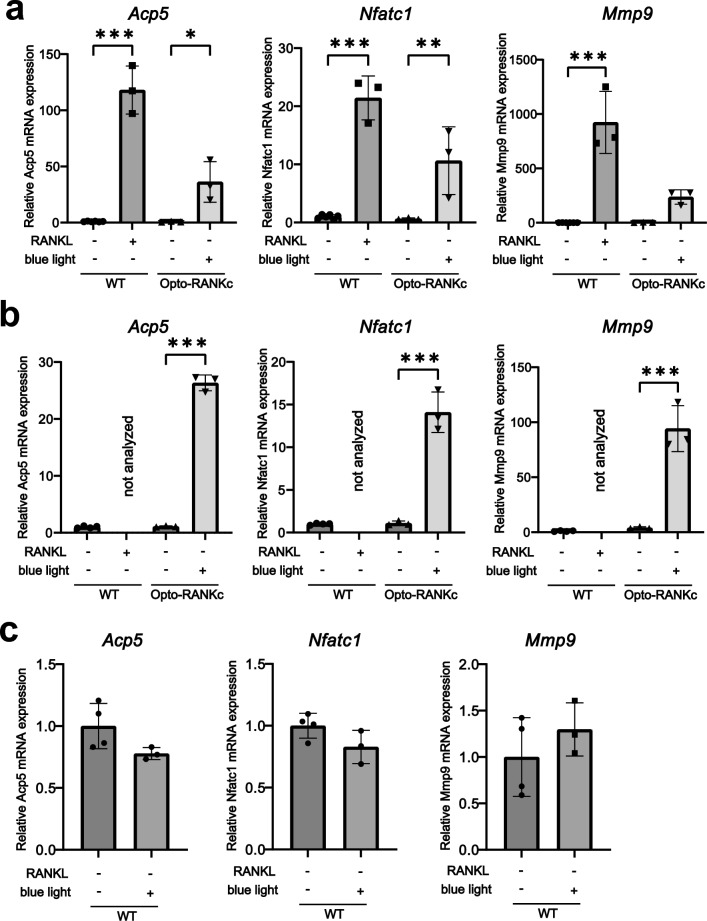
mRNA expression of genes related to osteoclast differentiation and function in Opto-RANKc cells induced using blue light. (**a**–**b**) Opto-RANKc cells were cultured with and without blue light exposure every 2 min. As a positive and negative control for differentiation, RAW264.7 cells (WT) were cultured in the presence and absence of RANKL, respectively. mRNA expression of *Acp5* (encoding TRAP), *Nfatc1*, and *Mmp9* was analyzed using quantitative PCR. Relative expression levels are normalized to the corresponding expression in RAW264.7 cells without RANKL and blue light. Relative gene expression in cells cultured for (**a**) 5 d and (**b**) 7 d. (**c**) RAW264.7 cells were exposed to blue light every 2 min for 7 d. The relative expression of *Acp5*, *Nfatc1*, and *Mmp9* is shown. All data represent mean ± SD from six (**a**) and four (**b**-**c**) independent experiments for WT without RANKL and blue light and three independent experiments for others. Data in (**a**) and (**b**) were analyzed using one-way analysis of variance followed by Tukey’s multiple comparisons test. Data in (**c**) were analyzed using two-tailed unpaired *t*-test for comparisons between two samples. *, *p* < 0.05; **, *p* < 0.01; ***, *p* < 0.001. RANK, receptor activator of nuclear factor-kB; RANKL, receptor activator of nuclear factor-kB ligand; WT, wild-type; TRAP, tartrate-resistant acid phosphatase; Acp5, acid phosphatase 5, tartrate resistant; Nfatc1, nuclear factor of activated T cells, cytoplasmic 1; Mmp9, matrix metallopeptidase 9.

To examine the gene expression pattern in differentiated Opto-RANKc cells, we performed quantitative PCR for three genes, acid phosphatase 5, tartrate resistant (*Acp5*, encoding TRAP), *Nfatc1*, and matrix metallopeptidase 9 (*Mmp9*), which are upregulated during osteoclast differentiation. RAW264.7 cells were differentiated into mature osteoclasts by RANKL in 5 d, and the expression of all three genes increased in mature osteoclasts (Fig. 5a). In Opto-RANKc cells, the expression of all three genes also increased significantly in 5 d and 7 d (Fig. 5a,b), although the extent of increase in expression was less in Opto-RANKc cells. This is consistent with the observations upon TRAP staining that the differentiation of Opto-RANKc cells is slower than that of RAW264.7 cells. We confirmed that blue light alone did not induce the expression of these osteoclast marker genes in RAW264.7 cells (Fig. 5c). These results suggested that Opto-RANKc cells are differentiated into mature osteoclasts based on the gene expression pattern.

### Pit-formation by differentiated Opto-RANKc cells

**Figure 6:**
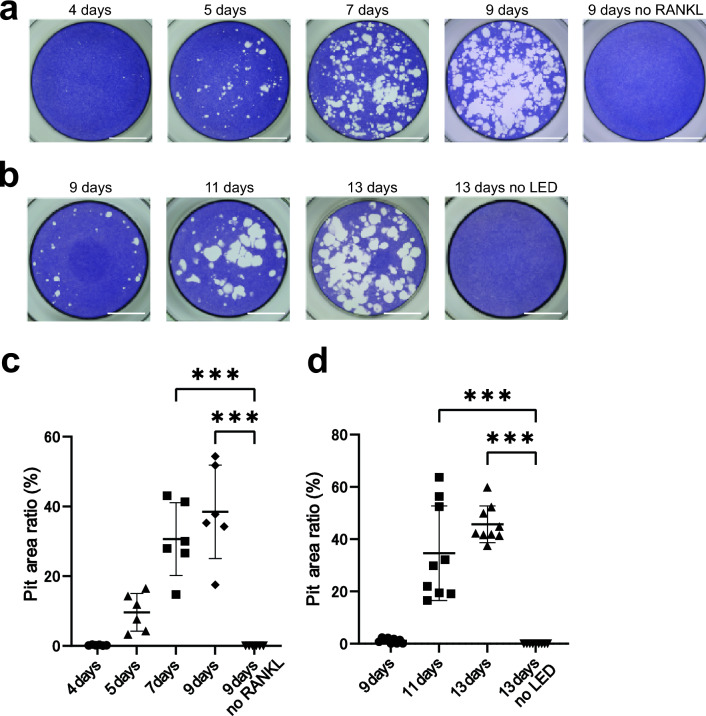
Measurement of bone-resorbing activity in CaP-coated plates. (**a**) RAW264.7 cells were cultured on CaP-coated 96-well plates in RANKL-containing medium for 4, 5, 7, and 9 d. Cells were also cultured in RANKL-free medium for 9 d, as a negative control. Representative images of the pit-formation are shown. Scale bar: 2 mm. (**b**) Opto-RANKc cells were cultured on collagen-coated plates for 4 d and passaged to CaP-coated plates. Cells were cultured for a total of 9, 11, and 13 d, with blue light exposure every 2 min. Cells were also cultured in the dark for a total of 13 d. Representative images of the pit-formation are shown. Scale bar: 2 mm. (**c**) Measurement of the pit areas in (**a**). (**d**) Measurement of the pit area in (**b**). The data in (**c**) and (**d**) represent the percentage of the pit area to that of the entire area in the well, represented as mean ± SD [*n* = 6 from six independent experiments for (**c**) and *n* = 9 from three independent experiments for (**d**)]. Data were analyzed using one-way analysis of variance followed by Tukey’s multiple comparisons test. ***, *p* < 0.001. RANKL, receptor activator of nuclear factor-kB ligand; CaP, calcium phosphate.

To examine whether differentiated Opto-RANKc cells have the function of bone-resorbing activity, we performed a pit-formation assay. Cells were cultured on CaP-coated plates, and bone-resorbing activity was quantified by measuring the pit area absorbed by osteoclasts. In the initial experiments, we compared four culture media and found that the amount of pit formation varied depending on the medium components (Supplementary Fig. S7). The CaP coat was stained with Trypan Blue, which had no effect on the CaP coat other than staining it blue (Supplementary Fig. S8). We cultured RAW264.7 and Opto-RANKc cells on the CaP-coated plates and then analyzed the pits formed by RAW264.7 cells differentiated with RANKL and those formed by Opto-RANKc cells differentiated with blue light (Fig. 6a,b). In both RAW264.7 and Opto-RANKc cells, the formation of many small pits was evident after 5 d and 9 d of culture, respectively (Fig. 6a,b), with the total pit size increasing thereafter (Fig. 6c,d). In both cases, the increase in pit area was accelerated at the periods after differentiation into large multinucleated cells. These results suggested that differentiated Opto-RANKc cells have a bone-resorbing function.

### Spatial control of CaP-absorption

**Figure 7:**
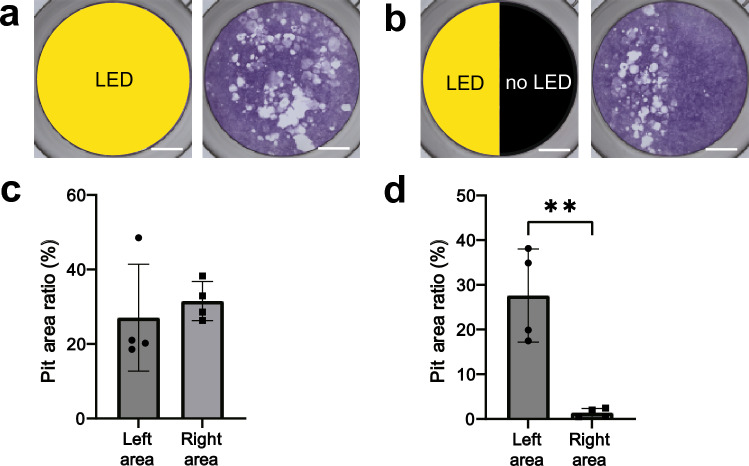
Spatial control of pit-formation. Opto-RANKc cells were cultured on CaP-coated 48-well plates with blue light exposure every 2 min, for 13 d, without passage. Representative image of pit-formation when (**a**) the entire well and (**b**) half of the well was illuminated with blue light. Scale bar: 2 mm. (**c** and **d**) Measurement of the pit area in (**a**) and (**b**), respectively. The wells were divided into two areas, left and right, and the pit area was measured. Left and right areas in (**b**) correspond to illuminated and unilluminated areas, respectively. The data show the area of the pits as a percentage of the area of the well halves, represented as mean ± SD from four independent experiments. Data were analyzed using two-tailed unpaired *t*-test. **, *p* < 0.01. CaP, calcium phosphate; RANK, receptor activator of nuclear factor-kB.

One of the advantages of optogenetic tools is the ability to spatially control cellular function. We examined whether CaP-absorption could be locally controlled by illuminating Opto-RANKc cells with blue light in a confined area. Half of a well in a 48-well plate was covered with aluminum foil to protect the area from light illumination. After Opto-RANKc cells were cultured in the well for 13 d, pit-formation analysis was performed. While pits were robustly formed on the illuminated side, almost no pits were formed on the light-protected side (Fig. 7a,b). Quantitative analysis also showed a sharp difference between the light-illuminated and light-protected sides (Fig. 7c,d). This result suggested that Opto-RANKc can be used for local bone resorption by controlling cell differentiation with targeted light illumination.

### Differentiation of Opto-RANKc cells in the presence of OPG

**Figure 8:**
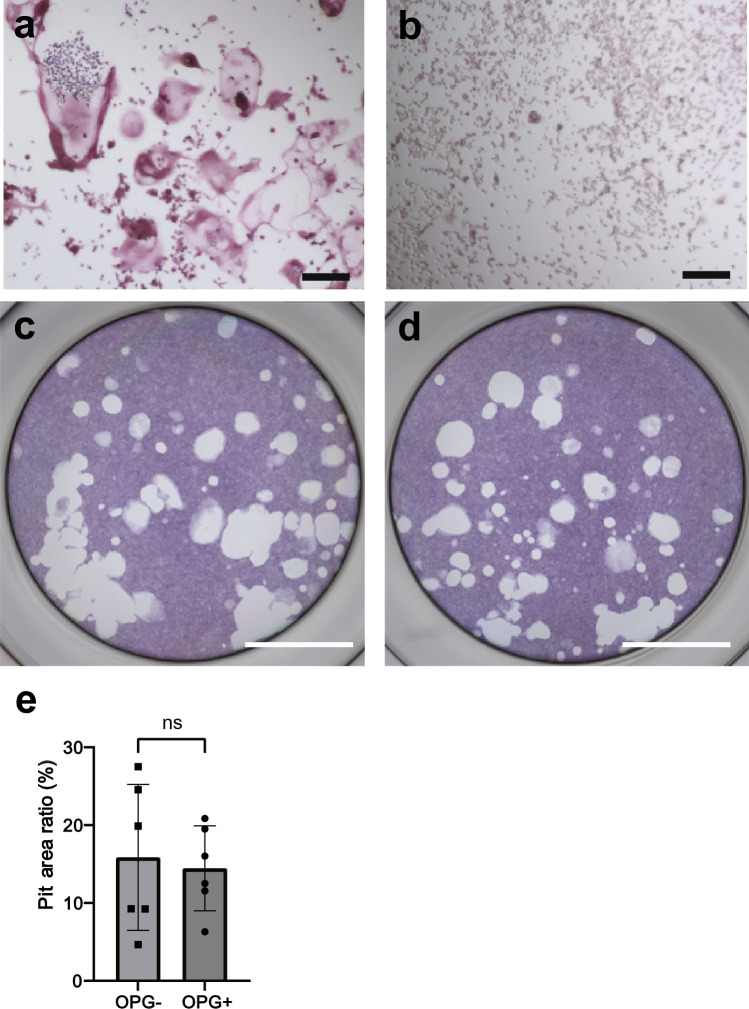
OPG-resistant differentiation of Opto-RANKc cells. (**a**-**b**) RAW264.7 cells were cultured in RANK-containing medium in the absence (**a**) and presence (**b**) of OPG. Cells were stained for TRAP and representative images are shown. OPG inhibited the RANKL-induced differentiation of RAW264.7 cells. Scale bar: 150 μm. (**c**-**d**) Opto-RANKc cells were cultured on CaP-coated 96-well plates in the absence (**c**) and presence (**d**) of OPG, with blue light exposure every 2 min, for 13 d, without passage. Representative images of pit-formation from six independent experiments. Scale bar: 2 mm. (**e**) Measurement of the pit area in (**c**) and (**d**). The data show the area of the pits as a percentage of the area of the entire well, represented as mean ± SD values from six independent experiments. Data were analyzed using two-tailed unpaired *t*-test. ns, not significant. RANK, receptor activator of nuclear factor-kB; TRAP, tartrate-resistant acid phosphatase; OPG, osteoprotegerin; CaP, calcium phosphate.

OPG is the decoy receptor for RANKL and functions as an inhibitor of osteoclastogenesis. Since Opto-RANKc functions in the absence of RANKL, OPG is expected to have no effect on Opto-RANKc-mediated osteoclast differentiation. First, the inhibitory effect of OPG on RANKL-induced differentiation of RAW264.7 cells was examined, and we confirmed that osteoclast differentiation was inhibited in the medium containing 500 ng/mL of OPG (Fig. 8a,b). To examine the influence of OPG on Opto-RANKc cells, we performed a pit-formation analysis in the presence or absence of OPG. Upon comparing the pits on the CaP-coated plates, we found similar amounts of pit area absorbed by light-induced differentiated Opto-RANKc cells in the presence and absence of OPG (Fig. 8c,d), with no significant difference between them (Fig. 8e). These results suggested that the light-induced differentiation of Opto-RANKc cells is not inhibited by OPG.

## Discussion

This study developed Opto-RANK, an optogenetic tool for osteoclast differentiation. Osteoclasts differentiated using Opto-RANKc displayed absorbing activity of the CaP substrate, thus making Opto-RANKc a unique optogenetic tool with specific and useful functions. The next crucial step is to test the Opto-RANK system in primary bone marrow cells to determine if it has any real potential for use in vivo, because RAW264.7 cells used in this study differentiate independently of M-CSF.

We engineered two Opto-RANK variants, Opto-RANKc and Opto-RANKm, owing to the uncertainty of whether RANK needs to function near the membrane for osteoclast differentiation. Opto-RANKc is a cytosolic photoswitch, whereas Opto-RANKm is a membrane-recruited photoswitch. Since some RAW264.7 clones expressing Opto-RANKc differentiated and formed large multinucleated osteoclasts upon light exposure in our study, we selected and extensively examined the Opto-RANKc cell line further. This, however, does not mean that Opto-RANKm was inferior, as Opto-RANKm cells had also differentiated into multinucleated cells but relatively smaller ones. The phenotype of the RAW264.7 cell line tends to change with passage^27^, which makes it difficult to ascertain whether the difference in differentiation could be attributed to the optogenetic constructs or repeated passage from a single cell. This highlights the need to examine more cloned lines to evaluate and compare the quality of the photoswitches. The least that can be concluded is that the RANK signal does not necessarily originate from the membrane vicinity and that cytoplasmic RANK homo-oligomerization is sufficient for osteoclast differentiation in RAW264.7 cells.

Compared with RANKL-induced differentiation of RAW264.7 cells, Opto-RANKc cells appear to require more time to be fully differentiated under our experimental condition of light exposure every 2 min. The delayed differentiation is consistent with the observation that our light illumination condition induced a lower level of initial MAPK phosphorylation than that observed in RANKL-stimulated RAW264.7 cells. Although increased light illumination increased the MAPK phosphorylation, it also increased toxicity to RAW264.7 cells. If toxicity could be reduced, Opto-RANKc cells could be differentiated at a similar rate as observed in the RANKL-induced differentiation of RAW264.7 cells. There could be several ways to improve the situation. For example, the expression level of Opto-RANKc could be elevated by changing the expression promoter or selecting clones with higher expression levels. Higher expression levels can induce more intense RANK signaling at the same light levels. Alternatively, changing the structure of Opto-RANKc in terms of alignment or domain spacing may facilitate rapid and efficient homo-oligomerization of the target molecule. The efficiency of Opto-RANKc oligomerization appears to be lower than that of the previously reported light-induced CRY2clust^31^.

Optogenetics-based therapies have begun to attract attention in recent years. Optogenetic therapy with channelrhodopsin partially restored visual function in a blind patient^36^. Optogenetic immunomodulation, in which optogenetic activation modulates innate^37–39^ and adaptive^40,41^ immunity, has been developed^1^. In addition, chimeric antigen receptor T cell-based immunotherapy combined with optogenetics has been developed, in which a functional chimeric antigen receptor could be formed upon photostimulation, resulting in spatiotemporal tumor cell killing^42,43^. The Opto-RANKc cells can be differentiated into osteoclasts morphologically and functionally. Differentiated Opto-RANKc cells have the ability to absorb the CaP substrate. Optical control allows for spatiotemporal induction of cell differentiation, as we observed from the local absorption of CaP. Therefore, Opto-RANKc cells may apply to cell therapies for heterotopic ossification. Cell therapy for vascular calcification has been proposed and demonstrated using bone marrow-derived osteoclasts^44^. Using bone marrow-derived osteoclasts to treat general ectopic calcification is not always appropriate due to the presence of OPG, an inhibitor of osteoclastogenesis. An alternative strategy to bypass OPG, in which chemical inducer-regulated RANK activation induces osteoclast differentiation, has been developed ^30^. This system is independent of RANKL and, therefore, resistant to OPG. Osteoclast differentiation by Opto-RANKc is also induced in the absence of RANKL and is not inhibited by OPG. The precise spatiotemporal control of Opto-RANKc activity makes it more suitable for application in cell therapy since such precise spatial control is difficult to achieve when using bone-marrow-derived or chemically regulated osteoclasts. After implantation of Opto-RANKc cells, blue light could be used to target cells in a confined area, thus making subsequent local absorption of the calcified substrate possible.

Opto-RANKc cells could be used for accelerating orthodontic tooth movements. Orthodontic tooth movement is a process of alveolar bone resorption on the compression side and new bone formation on the tension side^45^. Gene therapy using RANKL gene transfer to the periodontal tissues has been tested experimentally in rats and has shown higher efficacy than surgical methods in accelerating orthodontic treatment^46^. The differentiation of Opto-RANKc cells transplanted into the periodontal tissues could be controlled by blue light. The advantage of using Opto-RANKc cells is their ability to control bone resorption in a spatiotemporal manner using light. Osteoclast activity is coordinated with that of osteoblasts during development and homeostasis; thus, the coupling of osteoclasts and osteoblasts may be necessary for proper tooth movement. RANKL reverse signaling, a signal from osteoclasts to osteoblasts, promotes bone formation by secreting vesicular RANK from maturing osteoclasts and binding osteoblastic RANKL^47^. The communication between Opto-RANKc cells and osteoblasts has not yet been investigated and should be addressed in the future.

Optogenetic tools are useful for studying intracellular signaling because the signal can be freely controlled in a spatiotemporal manner. In Opto-RANK cells, the intensity, frequency, duration, and timing of the RANK signal can be controlled by blue light. Intracellular signaling is complex, and continuous RANK activation causes a dynamic temporal increase in MAPK phosphorylation and subsequent decrease. Using Opto-RANK, various parameters of RANK activation can be systematically tested, and the subsequent intracellular signaling and phenotypic outcome of cell differentiation can be analyzed. For spatial control, cellular functions could be examined at the subcellular level^24,25,48,49^. Osteoclasts are large cells formed by multiple cell fusions. The cytoplasmic motif of RANK is likely to regulate the actin cytoskeleton via Vav3, Rac1, and Cdc42 and be involved in the bone-resorbing function of differentiated osteoclasts^50^. The characteristic of osteoclast giant cells makes the subcellular analysis of osteoclasts more accessible. Since subcellular illumination with blue light could activate local RANK signaling, studying subcellular RANK function in cytoskeletal reorganization and bone resorption is challenging.

Opto-RANK will be useful beyond bone biology. The RANKL/RANK system has many functions in osteoclast differentiation, the immune system, mammary gland and hair follicle formation, body temperature regulation, muscle metabolism, and tumor development^51^. Furthermore, RANK is a member of the tumor necrosis factor receptor superfamily, which can bind tumor necrosis factors and play an essential role in the modulation of cellular functions, including immune regulation, programmed cell death, cell survival, and cellular differentiation^52,53^. There are 29 members of the tumor necrosis factor receptor superfamily, consisting of three distinct groups: death receptors, receptors with TRAF-interacting motif, and decoy receptors. The engineering of Opto-RANK in this study will extend the repertoire of optogenetic tools available to control other members of the tumor necrosis factor receptor superfamily with a TRAF-interacting motif, which regulates diverse biological functions such as the induction of cell survival and proliferation via the activation of the NF-kB family and various MAPK cascades.

The ability to manipulate RANK signaling at a subcellular level will help us understand the precise functions of RANK in osteoclast formation and function. Furthermore, the ability of Opto-RANK to differentiate preosteoclasts into mature osteoclasts will pave the way for new cellular therapies, such as the treatment of abnormal calcification diseases and dental orthodontics.

## Methods

### DNA constructs

*mCherry-CRY2clust* (plasmid no. 105624, Addgene, Watertown, MA, USA) was a gift from Dr. Won Do Heo^31^; *FLAG-TRAF6-wt* (plasmid no. 21624, Addgene) from Dr. John Kyriakis^54^; *OptoRaf* (plasmid no. 207163, Addgene) from Dr. Kai Zhang^55^. To generate *Opto-RANKm*, we performed plasmid constructions as follows: *GFP* was removed from *OptoRaf*, a silent mutation at an XhoI site was introduced into *CIBNx2* using recombinant PCR, and *CRY2* was replaced by the *CRY2clust* PCR fragment, with a silent mutation at a KpnI site amplified from *mCherry-CRY2clust*, resulting in *CRY2clust-mCherry-Raf1-P2A-CIBNcaax*. *Raf1* was replaced by the PCR fragment of the mouse *RANK* cytoplasmic domain (235–625 a.a. in Genbank ID NM_009399.5) amplified from a mouse thymus first-strand cDNA library. The *CRY2clust-mCherry-RANK-P2A-CIBNcaax* fragment was transferred into a pCAG vector containing a Woodchuck Hepatitis Virus post-transcriptional regulatory element^49^. To generate *Opto-RANKc*, *P2A-CIBNcaax* was removed from *Opto-RANKm* with a stop codon insertion in the 3'-untranslated region. To generate *GFP-TRAF6*, we replaced the *FLAG* tag in *FLAG-TRAF6-wt* with the *GFP* PCR fragment between the BamHI and EcoRI sites. The *TRAF6* is of murine origin. All cloned fragments were verified by sequencing.

The retroviral vectors were generated by inserting the PacI/NotI fragment of *Opto-RANKc* or *Opto-RANKm* into the pDON-5-neo vector (TaKaRa Bio, Tokyo, Japan), with the insertion of PacI and NotI restriction sites in the multiple cloning site.

The nucleotide and amino acid sequences of Opto-RANK are described in Supplementary Fig. S1. Opto-RANK expression vectors will be available soon from Addgene (http://www.addgene.org): *Opto-RANKc* (*CRY2clust-mCherry-RANK*, catalog no. 208612, Addgene) and *Opto-RANKm* (*CRY2clust-mCherry-RANK-P2A-CIBNcaax*, catalog no. 208613, Addgene).

### Cell culture and transfection

HEK293T cells were purchased from TaKaRa Bio (Shiga, Japan), and RAW264.7 cells were a kind gift from Dr. Suda, Showa University, Tokyo, Japan. Cells were maintained in the following media: for HEK293T, minimal essential medium (MEM) (catalog no. 2144225, Nacalai Tesque, Kyoto, Japan) supplemented with 10% (v/v) heat-inactivated fetal bovine serum (FBS); for RAW264.7, MEM supplemented with 10% (v/v) FBS, non-essential amino acid mixture (NEAA, Nacalai Tesque), and L-glutamine (L-GLU, Nacalai Tesque). HEK293T cells were seeded on glass-bottomed dishes (Greiner Bio-one, Kremsmünster, Austria) coated with collagen (Cellmatrix® Type IC; Nitta Gelatin, Osaka, Japan), 2 d before the experiments. The next day, plasmid transfection was performed using Lipofectamine™ 2000 (Thermo Fisher Scientific, Waltham, MA, USA), according to the manufacturer’s instructions, and the cells were analyzed 24–36 h after transfection.

### Generation of cell lines

Recombinant retroviruses were prepared using Retrovirus Packaging Kit Ampho (TaKaRa Bio), according to the manufacturer’s instructions. One day before transfection, HEK293T cells (1 × 10^6^ cells/dish) were plated in collagen I-coated 60-mm dishes (IWAKI, Tokyo, Japan), in Dulbecco’s modified Eagle’s medium (high-glucose, Nacalai Tesque) containing 10% FBS. The retroviral vectors and packaging plasmids, pGP and pE-ampho, were transfected into HEK293T using Lipofectamine™ 2000. The medium was replaced with fresh medium 24 h after transfection. After 2 d, the supernatants were filtrated through a 0.45-μm filter, and the recombinant retroviruses were concentrated using the Retro-X™ Concentrator (TaKaRa Bio), according to the manufacturer’s instructions. The viruses were suspended in Opti-MEM™ (Thermo Fisher Scientific). RAW264.7 cells were transduced with the concentrated viruses and polybrene (Nacalai Tesque). Six hours after transduction, the medium was replaced with fresh medium, and 2 d later, it was replaced with a culture medium containing G418 (Nacalai Tesque) (400–500 μg/mL) for > 7 d. We cultured single cells to establish cell lines (Opto-RANKc and Opto-RANKm cells) using limiting dilution of G418-selected cells in 96-well plates.

### Custom-built irradiation system

To deliver blue light (wavelength = 470 nm) to the bottom of the culture dishes, we used a custom-built irradiation system described previously^56^, with some modifications. An array of seven blue light-emitting diodes (LEDs) (LUXEON Rebel LXML-PB01-0040, Philips Lumileds Lighting, San Jose, CA, USA) was mounted in series on a board (281MCPCB LED Prototyping Board, Polymer Optics, Coventry, England). LED irradiation was controlled using a pulse generator (SEN-7203) with a current booster (SEG-3104) (both from Nihon Kohden, Tokyo, Japan). The power density of the LED arrays through a diffuser lens (Polymer Optics) was 0.9 mW mm^–2^ at the bottom of the culture dish.

### Induction of differentiation

Culture plates were left uncoated or coated with collagen, depending on the experiments. RAW264.7 cells were seeded into 96-well plates (3 × 10^3^ per well) for 5 d in a growth medium containing 50 ng/mL RANKL (FUJIFILM Wako Pure Chemicals, Osaka, Japan). Opto-RANKc and Opto-RANKm cells were seeded into 96-well plates (3 × 10^3^ per well) for 5 d and 7 d, with blue light (wavelength = 470 nm) exposure every 2 min, consisting of 12 cycles of 10 ms irradiation, with a 100-ms interval, unless stated otherwise. In 7-d culture, cells were replated into 96-well plates (at a density of 6 × 10^4^ cells per well) after 4 d of culture. The medium was changed every 2 d. For OPG inhibition experiments, OPG (Elabscience Biotechnology, Wuhan, China) was added to the culture medium at a concentration of 500 ng/mL.

### Live cell imaging and photoactivation

*Opto-RANKc* or *Opto-RANKm* containing *mCherry* was transfected into HEK293T cells with *GFP-TRAF6*. Prior to observations, the medium was replaced with Ringer’s solution [138 mM NaCl, 5.6 mM KCl, 2 mM CaCl_2_, 2 mM MgCl_2_, 9.4 mM D-glucose, 5 mM HEPES, and 2 mM sodium pyruvate (pH 7.4, adjusted with NaOH)]. Time-lapse images were obtained using a confocal laser scanning microscope (FV1200, Olympus, Tokyo, Japan) on an IX83 microscope equipped with 40 × /0.95 NA dry objective lenses and FV10-ASW software (Olympus). Photoactivation and imaging were performed every 5 s, at a scan rate of 10 μs per pixel (pixel size: 0.33 μm), with standard EGFP and DsRed2 settings (10% 488-nm argon laser power; 20% 559-nm diode laser power; DM 405/488/559/635 dichroic excitation, an SDM560 emission filter; 500–545 nm and 570–670 nm emission windows for EGFP and DsRed2, respectively) using the sequential line acquisition mode. The GFP and mCherry images were acquired using the EGFP and DsRed2 channels, respectively. The experiments were performed at room temperature.

### Tartrate-resistant acid phosphatase (TRAP) staining and measurement of TRAP activity

RAW264.7 or Opto-RANK cells were differentiated as described above. Cells in 96-well plates were fixed and stained with the TRAP/ALP Staining Kit (FUJIFILM Wako Pure Chemicals), according to the manufacturer’s instructions. For experiments shown in Fig. 4, cells were stained with 4',6-diamidino-2-phenylindole (0.5 μg/mL, Nacalai Tesque). Brightfield and fluorescence images of the cells were captured using BZ-X700 and BZ-X800 microscopes (Keyence, Osaka, Japan). TRAP activity in the cell lysate was assessed using the TRACP & ALP Assay Kit (TaKaRa Bio), according to the manufacturer’s instructions. Absorbance was measured at 405 nm using a Multiskan Sky microplate spectrophotometer (Thermo Fisher Scientific).

### Western blot analysis

RAW264.7 or Opto-RANKc cells (4 × 10^5^ cells/well) were seeded in a 35-mm cell culture dish containing MEM supplemented with 10% heat-inactivated FBS, NEAA mixture, and L-GLU at 2 d before the cell lysis experiments. The following day, the medium was replaced with MEM supplemented with 1% heat-inactivated FBS, NEAA, and L-GLU, while on the day of the cell lysis experiments, it was replaced with MEM supplemented with 0.5% heat-inactivated FBS, NEAA, and L-GLU. For RANKL-stimulation experiments, the medium for RAW264.7 cells was replaced with MEM supplemented with 0.5% heat-inactivated FBS, NEAA, and L-GLU containing 50 ng/mL of RANKL (FUJIFILM Wako Pure Chemicals). For the LED-stimulation experiments, cells were exposed to blue light (wavelength = 470 nm) at the rate of every 10 s, 30 s, or 2 min, with each exposure consisting of 12 cycles of 10 ms irradiation and 100-ms intervals. Immunoblots and immunoblot detection were performed as described^57^, with some modifications. Briefly, at each time-point, cells were lysed in 2 × sodium dodecyl sulfate sample buffer [125 mM Tris–HCl pH 6.8, 4% (w/v) sodium dodecyl sulfate, 10% (w/v) sucrose, 0.01% (w/v) bromophenol blue, 5% (v/v) 2-mercaptoethanol]. Proteins were separated using sodium dodecyl sulfate–polyacrylamide gel electrophoresis and blotted onto polyvinylidene fluoride membranes (Merck, Darmstadt, Germany) using a Trans-Blot device (Bio-Rad, Richmond, CA, USA). Blots were blocked for 1 h at room temperature with 2% bovine serum albumin (Nacalai Tesque) in Tris-buffered saline with Tween-20 (Nacalai Tesque). Antibody reactions were performed at room temperature with primary antibodies diluted in Can Get Signal Solution 1 (TOYOBO, Osaka, Japan) for 1 h and corresponding horseradish peroxidase-conjugated secondary antibodies diluted in Can Get Signal Solution 2 (TOYOBO) for 1 h. Immunoreactive bands were visualized using ImmunoStar® Zeta (FUJIFILM Wako Pure Chemicals), and chemiluminescence signals were detected using a ChemiDoc™ MP system (Bio-Rad). We used WB Stripping Solution Strong (Nacalai Tesque) to strip the antibody from the blotting membrane. Blotted membranes were prepared for each antibody reaction, except for the anti-β-actin antibody, where the antibody-stripped membrane was used.

### Antibodies

The following commercial antibodies from Cell Signaling Technology were used : rabbit anti-p38 MAPK antibody (catalog no. 8690, 1:4000 dilution); rabbit anti-phospho-p38 MAPK antibody (catalog no. 4511, 1:2000 dilution); mouse anti-p44/42 MAPK (Erk1/2) antibody (catalog no. 4696, 1:2000 dilution); rabbit anti-phospho-p44/42 MAPK (Erk1/2) antibody (catalog no. 4370, 1:4000 dilution); mouse anti-β-actin antibody (catalog no. 3700, 1:4000 dilution); and peroxidase-conjugated goat anti-rabbit IgG (catalog no. 7074, 1:4000 dilution). Peroxidase-conjugated sheep anti-mouse IgG antibody was obtained from Cytiva (Tokyo, Japan; catalog no. NA931, 1:2000 dilution). Peroxidase-conjugated donkey anti-rat IgG antibody was obtained from Jackson ImmunoResearch (West Grove, PA, USA; catalog no. 712-035-150, 1:5000 dilution). Rat anti-mCherry antibody was obtained from Thermo Fisher Scientific (catalog no. M11217, 1:1000 dilution).

### Quantitative RT-PCR analysis

RAW264.7 and Opto-RANKc cells were differentiated in 96-well plates, as described above. For the 7-d culture, Opto-RANKc cells were replated after 4 d of culture. Total RNA isolation and cDNA synthesis were performed using the SuperPrep II Cell Lysis & RT Kit for qPCR (TOYOBO), according to the manufacturer’s instructions. Quantitative real-time PCR was performed using the StepOnePlus Real-Time PCR System (Applied Biosystems, Foster City, CA, USA). The mRNA expression level was normalized to that of *Gapdh* expression. The following primer sequences were used: 5′-CGTCTCTGCACAGATTGCAT-3′ and 5′-AAGCGCAAACGGTAGTAAGG-3′ for *Acp5* (TRAP); 5′-TGGTCACTGGGGAACATACA-3′ and 5′-CACCAGGGGACAGCATTATT-3′ for *Nfatc1*; and 5′-CTGGACAGCCAGACACTAAAG-3′ and 5′-CTCGCGGCAAGTCTTCAGAG-3′ for *Mmp9*.

### Preparation of CaP-coated plates and pit-formation assay

The CaP-coated plates were prepared as described previously^58^, with some modifications. Briefly, 1 mL of 0.12 M Na_2_HPO_4_ (50 mM Tris–HCl, pH 7.4) and 1 mL of 0.2 M CaCl_2_ (50 mM Tris–HCl, pH 7.4) were pre-incubated in a water bath maintained at 37℃, and mixed. The resulting CaP slurry was washed thrice with 10 mL of sterile water and suspended in 15 mL of sterile water. The suspension (80 and 250 μL) was added to each well of 96- and 48-well plates, respectively. The plates were dried at 37 ℃ for 24 h and heated at 80 ℃ for 3 h. The CaP-coated plates were used without any additional coating procedures.

For the pit-formation assay, Dulbecco’s modified Eagle’s medium/Ham’s F-12 without Phenol Red (catalog no. 0517715, Nacalai Tesque) supplemented with 10% FBS was used as the medium component unless otherwise stated. RAW264.7 cells (3,000 cells/well) were seeded in CaP-coated 96-well plates and cultured in the presence or absence of RANKL for 4, 5, 7, and 9 d. For Opto-RANKc cells, the cell culture condition varied depending on the following experiments. For Fig. 6b,d, cells were plated into collagen-coated 96-well plates (3,000 cells/well) for 4 d, replated in CaP-coated 96-well plates (60,000 cells/well), and cultured for further 5, 7, and 9 d. For Figs. 7, 8, and Supplementary Fig. S7, cells (3,000 or 9,000 cells/well) were seeded in CaP-coated 96- or 48-well plates, respectively, and cultured for 13 d without passage. For Fig. 7b,d, half of the well bottoms were covered with aluminum foil to protect the cells from LED light.

To observe the pits formed at the bottom of the well, we removed the medium and incubated the cells with 10% sodium hypochlorite solution (Nacalai Tesque) for 5 min at room temperature. The wells were washed twice with distilled water and dried at room temperature. To visualize the CaP-coating, we stained the wells with Trypan Blue (Thermo Fisher Scientific) for 10 min. After washing with distilled water twice, the wells were dried. Pit formation was observed and analyzed using a BZ-X700/800 microscope (Keyence).

### Statistical analyses

Statistical analyses for comparisons between two samples were performed using a two-tailed unpaired *t-*test (Figs. 5c, 7c, 7d, 8e, and Supplementary Fig. S6c) and a two-tailed paired *t*-test (Supplementary Fig. S8e). Comparisons between more than two samples were performed using a one-way analysis of variance followed by Tukey’s test (Figs. 4d,e,f, 5a,b, 6c,d, and S7e), using Prism 10.0.1 (GraphPad Software, San Diego, CA, USA). *p*-values < 0.05 were considered statistically significant. Graphs were generated using the Prism 10 software.

### Supplementary Information

Supplementary Information.

## Data Availability

The datasets used and/or analyzed during the current study are available from the corresponding author on reasonable request.
