# Supplementary material for: Development of an optogenetics tool, Opto-RANK, for control of osteoclast differentiation using blue light
Source: Sci Rep. 2024 Jan 19;14:1749. doi: 10.1038/s41598-024-52056-w (PMC10799070; doi:10.1038/s41598-024-52056-w)
Supplement: Supplementary file 1 — Supplementary Information. [file 41598_2024_52056_MOESM1_ESM.pdf]

Supplementary information

Development of an optogenetics tool, Opto-RANK, for control of osteoclast differentiation using blue light

Aiko Takada, Toshifumi Asano, Ken-ichi Nakahama, Takashi Ono, Takao Nakata*, Tomohiro Ishii*

*Corresponding authors:
Takao Nakata, E-mail: info.cbio@tmd.ac.jp
Tomohiro Ishii, E-mail: ishii.t.aw@m.titech.ac.jp

Supplementary Figure S1

Opto-RANKc

CRY2

Clust enhancing peptide

mCherry

RANK cytoplasmic fragment

Nucleotide sequence

ATGAAGATGGACAAAAAGACCATCGTCTGGTTTCGGAGAGATTTGAGAATAGAAGAT
AATCCCGCGCTCGCCGCCGCGGCCCACGAGGGTTCCGTCTTCCCCGTTTTCATTTGG
TGTCCTGAAGAAGAAGGCCAGTTTTATCCCGGAAGGGCCTCTAGGTGGTGGATGAA
GCAAAGTCTGGCCCATCTTAGCCAGTCACTGAAAGCACTGGGCAGTGATCTTACCCT
GATCAAGACACACAATACCATCTCTGCCATCCTCGACTGCATCAGGGTGACCGGCGC
AACGAAAGTCGTGTTTAACCACCTGTACGATCCAGTTAGTCTGGTGCGCGACCACAC
TGTGAAGGAGAAGCTGGTGGAACGGGGGATCAGTGTGCAGAGCTACAACGGGGAC
CTTCTGTACGAGCCATGGGAGATCTATTGCGAGAAAGGGAAACCGTTCACCTCCTTC
AACAGTTACTGGAAGAAATGTTTGGATATGTCAATAGAGTCCGTTATGTTGCCCCCTC
CCTGGAGACTGATGCCGATTACTGCTGCTGCAGAGGCCATCTGGGCCTGCTCCATCG
AGGAACTCGGTCTGGAAAATGAAGCAGAAAAGCCAAGCAATGCACTTCTCACTAGA
GCCTGGAGCCCCGGCTGGTCTAATGCCGACAAGCTGCTTAACGAGTTCATCGAAAA
ACAACTGATTGACTACGCGAAGAACTCCAAGAAAGTGGTAGGTAACTCAACTAGCT
TGCTCTCTCCATATCTCCATTTTGGCGAGATTTCTGTCCGCCATGTATTTCAGTGCGCT
CGGATGAAACAGATTATCTGGGCTCGCGATAAAAACAGCGAAGGCGAAGAAAGCGC
CGATCTGTTCCTGCGAGGGATCGGACTTCGGGAATACTCCCGGTATATATGTTTCAAC
TTTCCATTCACACACGAGCAGAGTCTGTTGTCCCACCTCAGGTTCTTCCCCTGGGAC
GCCGATGTCGACAAATTCAAGGCATGGAGACAGGGAAGGACAGGATACCCACTCGT
GGATGCTGGCATGAGAGAGCTCTGGGCTACAGGCTGGATGCACAACCGCATCCGGG
TAATCGTGTCCTCATTTGCTGTCAAGTTTCTGCTCCTGCCTTGGAAATGGGGAATGAA
GTACTTTTGGGATACCCTTCTCGACGCCGACTTGGAGTGTGACATTCTGGGATGGCA
ATATATTAGCGGGTCAATTCCTGACGGCCATGAGTTGGACAGGTTGGACAATCCGGC
CTTGCAGGGAGCTAAGTATGATCCCGAAGGAGAGTATATTCGACAGTGGCTCCCCGA
GCTGGCCCGACTTCCTACGGAGTGGATTCACCATCCTTGGGACGCACCACTGACAGT
GCTCAAGGCAAGCGGGGTGGAGCTGGGCACCAATTACGCTAAGCCTATAGTTGATAT
AGATACAGCACGCGAGCTGCTGGCTAAAGCGATCTCTCGCACTCGGGAGGCGCAGA
TTATGATCGGTGCTGCCGCCCGGGATCCACCGGATCTAGATAACGAATTCGATAGTGC

TGGTAGTGCTGGTAGTGCTGGTACTAGTATGGTGAGCAAGGGCGAGGAGGATAACAT
GGCCATCATCAAGGAGTTCATGCGCTTCAAGGTGCACATGGAGGGCTCCGTGAACG
GCCACGAGTTCGAGATCGAGGGCGAGGGCGAGGGCCGCCCCTACGAGGGCACCCA
GACCGCCAAGCTGAAGGTGACCAAGGGTGGCCCCCTGCCCTTCGCCTGGGACATCC
TGTCCCCTCAGTTCATGTACGGCTCCAAGGCCTACGTGAAGCACCCCGCCGACATCC
CCGACTACTTGAAGCTGTCCTTCCCCGAGGGCTTCAAGTGGGAGCGCGTGATGAACT
TCGAGGACGGCGGCGTGGTGACCGTGACCCAGGACTCCTCCCTGCAGGACGGCGA
GTTCATCTACAAGGTGAAGCTGCGCGGCACCAACTTCCCCTCCGACGGCCCCGTAAT
GCAGAAGAAGACCATGGGCTGGGAGGCCTCCTCCGAGCGGATGTACCCCGAGGACG
GCGCCCTGAAGGGCGAGATCAAGCAGAGGCTGAAGCTGAAGGACGGCGGCCACTA
CGACGCTGAGGTCAAGACCACCTACAAGGCCAAGAAGCCCGTGCAGCTGCCCGGC
GCCTACAACGTCAACATCAAGTTGGACATCACCTCCCACAACGAGGACTACACCATC
GTGGAACAGTACGAACGCGCCGAGGGCCGCCACTCCACCGGCGGCATGGACGAGCT
GTACAAGTCCGGACTCAGATCTCGAGTGGGTGGATCTGGAGGTTCATACTACAGGAA
GGGAGGGAAAGCGCTGACAGCTAATTTGTGGAATTGGGTCAATGATGCTTGCAGTAG
TCTAAGTGGAAATAAGGAGTCCTCAGGGGACCGTTGTGCTGGTTCCCACTCGGCAA
CCTCCAGTCAGCAAGAAGTGTGTGAAGGTATCTTACTAATGACTCGGGAGGAGAAG
ATGGTTCCAGAAGACGGTGCTGGAGTCTGTGGGCCTGTGTGTGCGGCAGGTGGGCC
CTGGGCAGAAGTCAGAGATTCTAGGACGTTCACACTGGTCAGCGAGGTTGAGACGC
AAGGAGACCTGTCGAGGAAGATTCCCACAGAGGATGAGTACACGGACCGGCCCTCG
CAGCCTTCGACTGGTTCACTGCTCCTAATCCAGCAGGGAAGCAAATCTATACCCCCAT
TCCAGGAGCCCCTGGAAGTGGGGGAGAACGACAGTTTAAGCCAGTGTTTCACCGGG
ACTGAAAGCACGGTGGATTCTGAGGGCTGTGACTTCACTGAGCCTCCGAGCAGAAC
TGACTCTATGCCCGTGTCCCCTGAAAAGCACCTGACAAAAGAAATAGAAGGTGACA
GTTGCCTCCCCTGGGTGGTCAGCTCCAACTCAACAGATGGCTACACAGGCAGTGGG
AACACTCCTGGGGAGGACCATGAACCCTTTCCAGGGTCCCTGAAATGTGGACCATTG
CCCCAGTGTGCCTACAGCATGGGCTTTCCCAGTGAAGCAGCAGCCAGCATGGCAGA
GGCGGGAGTACGGCCCCAGGACAGGGCTGATGAGAGGGGAGCCTCAGGGTCCGGG
AGCTCCCCCAGTGACCAGCCACCTGCCTCTGGGAACGTGACTGGAAACAGTAACTC
CACGTTCATCTCTAGCGGGCAGGTGATGAACTTCAAGGGTGACATCATCGTGGTGTA
TGTCAGCCAGACCTCGCAGGAGGGCCCGGGTTCCGCAGAGCCCGAGTCGGAGCCC
GTGGGCCGCCCTGTGCAGGAGGAGACGCTGGCACACAGAGACTCCTTTGCGGGCAC
CGCGCCGCGCTTCCCCGACGTCTGTGCCACCGGGGCTGGGCTGCAGGAGCAGGGGG
CACCCCGGCAGAAGGACGGGACATCGCGGCCGGTGCAGGAGCAGGGTGGGGCGCA
GACTTCACTCCATACCCAGGGGTCCGGACAATGTGCAGAAGGAGGTTCAGGTGGAT
CTGGTACCTGA

Amino acid sequence

MKMDKKTIVWFRRDLRIEDNPALAAAAHEGSVFPVFIWCPEEEGQFYPGRASRWWMK
QSLAHLSQSLKALGSDLTLIKTHNTISAILDCIRVTGATKVVFNHLYDPVSLVRDHTVKE
KLVERGISVQSYNGDLLYEPWEIYCEKGKPFTSFNSYWKKCLDMSIESVMLPPPWRLMP
ITAAAEAIWACSIEELGLENEAEKPSNALLTRAWSPGWSNADKLLNEFIEKQLIDYAKNS
KKVVGNSTSLLSPYLHFGEISVRHVFQCARMKQIIWARDKNSEGEESADLFLRGIGLRE
YSRYICFNFPFTHEQSLLSHLRFFPWDADVDKFKAWRQGRTGYPLVDAGMRELWATGW
MHNRIRVIVSSFAVKFLLLPWKWGMKYFWDTLLDADLECDILGWQYISGSIPDGHELD
RLDNPALQGAKYDPEGEYIRQWLPELARLPTEWIHHPWDAPLTVLKASGVELGTNYAK
PIVDIDTARELLAKAISRTREAQIMIGAAARDPPDLDNEFDSAGSAGSAGTSMVSKGEED
NMAIIKEFMRFKVHMEGSVNGHEFEIEGEGEGRPYEGTQTAKLKVTKGGPLPFAWDILS
PQFMYGSKAYVKHPADIPDYLKLSFPEGFKWERVMNFEDGGVVTVTQDSSLQDGEFIY
KVKLRGTNFPSDGPVMQKKTMGWEASSERMYPEDGALKGEIKQRLKLKDGGHYDAE
VKTTYKAKKPVQLPGAYNVNIKLDITSHNEDYTIVEQYERAEGRHSTGGMDELYKSGL
RSRVGGSGGSYYRKGGKALTANLWNWVNDACSSLSGNKESSGDRCAGSHSATSSQQE
VCEGILLMTREEKMVPEDGAGVCGPVCAAGGPWAEVRDSRTFTLVSEVETQGDLSRKI
PTEDEYTDRPSQPSTGSLLLIQQGSKSIPPFQEPLEVGENDSLSQCFTGTESTVDSEGCDF
TEPPSRTDSMPVSPEKHLTKEIEGDSCLPWVVSSNSTDGYTGSGNTPGEDHEPFPGSLKC
GPLPQCAYSMGFPSEAAASMAEAGVRPQDRADERGASGSGSSPSDQPPASGNVTGNSN
STFISSGQVMNFKGDIIVVYVSQTSQEGPGSAEPESEPVGRPVQEETLAHRDSFAGTAPR
FPDVCATGAGLQEQGAPRQKDGTSRPVQEQGGAQTSLHTQGSGQCAEGGSGGSGT*

Opto-RANKm

CRY2 Clust enhancing peptide
mCherry
RANK cytoplasmic domain 235-625 (Uniprot)
P2A CIBN CAAX

Nucleotide sequence

ATGAAGATGGACAAAAAGACCATCGTCTGGTTTCGGAGAGATTTGAGAATAGAAGAT
AATCCCGCGCTCGCCGCCGCGGCCCACGAGGGTTCCGTCTTCCCCGTTTTCATTTGG
TGTCCTGAAGAAGAAGGCCAGTTTTATCCCGGAAGGGCCTCTAGGTGGTGGATGAA
GCAAAGTCTGGCCCATCTTAGCCAGTCACTGAAAGCACTGGGCAGTGATCTTACCCT
GATCAAGACACACAATACCATCTCTGCCATCCTCGACTGCATCAGGGTGACCGGCGC

AACGAAAGTCGTGTTTAACCACCTGTACGATCCAGTTAGTCTGGTGCGCGACCACAC
TGTGAAGGAGAAGCTGGTGGAACGGGGGATCAGTGTGCAGAGCTACAACGGGGAC
CTTCTGTACGAGCCATGGGAGATCTATTGCGAGAAAGGGAAACCGTTCACCTCCTTC
AACAGTTACTGGAAGAAATGTTTGGATATGTCAATAGAGTCCGTTATGTTGCCCCCTC
CCTGGAGACTGATGCCGATTACTGCTGCTGCAGAGGCCATCTGGGCCTGCTCCATCG
AGGAACTCGGTCTGGAAAATGAAGCAGAAAAGCCAAGCAATGCACTTCTCACTAGA
GCCTGGAGCCCCGGCTGGTCTAATGCCGACAAGCTGCTTAACGAGTTCATCGAAAA
ACAACTGATTGACTACGCGAAGAACTCCAAGAAAGTGGTAGGTAACTCAACTAGCT
TGCTCTCTCCATATCTCCATTTTGGCGAGATTTCTGTCCGCCATGTATTTCAGTGCGCT
CGGATGAAACAGATTATCTGGGCTCGCGATAAAAACAGCGAAGGCGAAGAAAGCGC
CGATCTGTTCCTGCGAGGGATCGGACTTCGGGAATACTCCCGGTATATATGTTTCAAC
TTTCCATTCACACACGAGCAGAGTCTGTTGTCCCACCTCAGGTTCTTCCCCTGGGAC
GCCGATGTCGACAAATTCAAGGCATGGAGACAGGGAAGGACAGGATACCCACTCGT
GGATGCTGGCATGAGAGAGCTCTGGGCTACAGGCTGGATGCACAACCGCATCCGGG
TAATCGTGTCCTCATTTGCTGTCAAGTTTCTGCTCCTGCCTTGGAAATGGGGAATGAA
GTACTTTTGGGATACCCTTCTCGACGCCGACTTGGAGTGTGACATTCTGGGATGGCA
ATATATTAGCGGGTCAATTCCTGACGGCCATGAGTTGGACAGGTTGGACAATCCGGC
CTTGCAGGGAGCTAAGTATGATCCCGAAGGAGAGTATATTCGACAGTGGCTCCCCGA
GCTGGCCCGACTTCCTACGGAGTGGATTCACCATCCTTGGGACGCACCACTGACAGT
GCTCAAGGCAAGCGGGGTGGAGCTGGGCACCAATTACGCTAAGCCTATAGTTGATAT
AGATACAGCACGCGAGCTGCTGGCTAAAGCGATCTCTCGCACTCGGGAGGCGCAGA
TTATGATCGGTGCTGCCGCCCGGGATCCACCGGATCTAGATAACGAATTCGATAGTGC
TGGTAGTGCTGGTAGTGCTGGTACTAGTATGGTGAGCAAGGGCGAGGAGGATAACAT
GGCCATCATCAAGGAGTTCATGCGCTTCAAGGTGCACATGGAGGGCTCCGTGAACG
GCCACGAGTTCGAGATCGAGGGCGAGGGCGAGGGCCGCCCCTACGAGGGCACCCA
GACCGCCAAGCTGAAGGTGACCAAGGGTGGCCCCCTGCCCTTCGCCTGGGACATCC
TGTCCCCTCAGTTCATGTACGGCTCCAAGGCCTACGTGAAGCACCCCGCCGACATCC
CCGACTACTTGAAGCTGTCCTTCCCCGAGGGCTTCAAGTGGGAGCGCGTGATGAACT
TCGAGGACGGCGGCGTGGTGACCGTGACCCAGGACTCCTCCCTGCAGGACGGCGA
GTTCATCTACAAGGTGAAGCTGCGCGGCACCAACTTCCCCTCCGACGGCCCCGTAAT
GCAGAAGAAGACCATGGGCTGGGAGGCCTCCTCCGAGCGGATGTACCCCGAGGACG
GCGCCCTGAAGGGCGAGATCAAGCAGAGGCTGAAGCTGAAGGACGGCGGCCACTA
CGACGCTGAGGTCAAGACCACCTACAAGGCCAAGAAGCCCGTGCAGCTGCCCGGC
GCCTACAACGTCAACATCAAGTTGGACATCACCTCCCACAACGAGGACTACACCATC
GTGGAACAGTACGAACGCGCCGAGGGCCGCCACTCCACCGGCGGCATGGACGAGCT
GTACAAGTCCGGACTCAGATCTCGAGTGGGTGGATCTGGAGGTTCATACTACAGGAA

GGGAGGGAAAGCGCTGACAGCTAATTTGTGGAATTGGGTCAATGATGCTTGCAGTAG
TCTAAGTGGAAATAAGGAGTCCTCAGGGGACCGTTGTGCTGGTTCCCACTCGGCAA
CCTCCAGTCAGCAAGAAGTGTGTGAAGGTATCTTACTAATGACTCGGGAGGAGAAG
ATGGTTCCAGAAGACGGTGCTGGAGTCTGTGGGCCTGTGTGTGCGGCAGGTGGGCC
CTGGGCAGAAGTCAGAGATTCTAGGACGTTCACACTGGTCAGCGAGGTTGAGACGC
AAGGAGACCTGTCGAGGAAGATTCCCACAGAGGATGAGTACACGGACCGGCCCTCG
CAGCCTTCGACTGGTTCACTGCTCCTAATCCAGCAGGGAAGCAAATCTATACCCCCAT
TCCAGGAGCCCCTGGAAGTGGGGGAGAACGACAGTTTAAGCCAGTGTTTCACCGGG
ACTGAAAGCACGGTGGATTCTGAGGGCTGTGACTTCACTGAGCCTCCGAGCAGAAC
TGACTCTATGCCCGTGTCCCCTGAAAAGCACCTGACAAAAGAAATAGAAGGTGACA
GTTGCCTCCCCTGGGTGGTCAGCTCCAACTCAACAGATGGCTACACAGGCAGTGGG
AACACTCCTGGGGAGGACCATGAACCCTTTCCAGGGTCCCTGAAATGTGGACCATTG
CCCCAGTGTGCCTACAGCATGGGCTTTCCCAGTGAAGCAGCAGCCAGCATGGCAGA
GGCGGGAGTACGGCCCCAGGACAGGGCTGATGAGAGGGGAGCCTCAGGGTCCGGG
AGCTCCCCCAGTGACCAGCCACCTGCCTCTGGGAACGTGACTGGAAACAGTAACTC
CACGTTCATCTCTAGCGGGCAGGTGATGAACTTCAAGGGTGACATCATCGTGGTGTA
TGTCAGCCAGACCTCGCAGGAGGGCCCGGGTTCCGCAGAGCCCGAGTCGGAGCCC
GTGGGCCGCCCTGTGCAGGAGGAGACGCTGGCACACAGAGACTCCTTTGCGGGCAC
CGCGCCGCGCTTCCCCGACGTCTGTGCCACCGGGGCTGGGCTGCAGGAGCAGGGGG
CACCCCGGCAGAAGGACGGGACATCGCGGCCGGTGCAGGAGCAGGGTGGGGCGCA
GACTTCACTCCATACCCAGGGGTCCGGACAATGTGCAGAAGGAGGTTCAGGTGGAT
CTGGTACCGGAAGCGGAGCTACTAACTTCAGCCTGCTGAAGCAGGCTGGAGACGTG
GAGGAGAACCCTGGACCTGCTCCAGGATCCGACTCTGCTGGCTCAGCCGGAATGAA
CGGGGCTATAGGAGGCGACCTCTTGTTGAACTTTCCTGACATGTCAGTCTTGGAACG
ACAGCGCGCCCACCTGAAGTACTTGAATCCCACCTTCGATAGCCCACTGGCTGGCTT
TTTCGCCGATTCATCAATGATCACAGGGGGAGAAATGGATTCCTACCTCAGCACTGC
AGGACTGAATCTGCCGATGATGTACGGAGAAACGACTGTGGAGGGCGATTCCCGCC
TGTCCATTAGCCCAGAAACCACTCTGGGCACCGGGAACTTTAAGGCTGCCAAATTTG
ATACTGAAACTAAGGATTGCAACGAAGCCGCAAAAAAAATGACTATGAACCGGGAT
GATCTGGTGGAGGAGGGTGAGGAGGAGAAAAGCAAAATAACAGAGCAAAACAATG
GCAGCACCAAGAGCATCAAGAAAATGAAGCATAAGGCCAAGAAAGAAGAAAACAA
CTTCAGTAATGATTCTAGCAAAGTGACAAAAGAGCTCGAAAAAACCGATTACATCGC
CCCGGGTTCTGATAGCGCAGGCAGTGCTGGTATGAACGGAGCTATTGGAGGAGATCT
GTTGCTGAACTTCCCTGATATGTCTGTCCTTGAGCGACAACGCGCACACCTCAAGTA
TCTGAATCCTACCTTTGACAGCCCTCTGGCCGGATTCTTTGCCGACTCATCCATGATT
ACAGGGGGCGAAATGGACTCCTATCTCAGCACAGCCGGGCTCAACCTCCCCATGATG

TATGGCGAGACGACCGTCGAAGGCGACTCTAGGCTCAGCATCAGCCCGGAAACTAC
CTTGGGAACAGGGAACTTCAAGGCTGCCAAGTTCGACACCGAAACAAAAGACTGC
AATGAAGCTGCCAAGAAGATGACAATGAATAGGGACGATTTGGTCGAGGAAGGAGA
AGAAGAGAAGAGCAAGATTACAGAACAAAATAACGGCTCCACTAAGTCCATCAAAA
AGATGAAGCACAAAGCCAAAAAGGAGGAGAATAACTTCAGCAACGATAGCTCCAA
GGTGACCAAGGAGCTGGAAAAGACCGACTATATCACCGGTGGAGGCTCTTCTGCTG
GTGGTTCAGCTGGAGGAAGTGCTGGTGGTAAAAAGAAGAAAAAGAAGTCAAAGAC
AAAGTGTGTAATTATGTAA

Amino acid sequence

MKMDKKTIVWFRRDLRIEDNPALAAAAHEGSVFPVFIWCPEEEGQFYPGRASRWWMK
QSLAHLSQSLKALGSDLTLIKTHNTISAILDCIRVTGATKVVFNHLYDPVSLVRDHTVKE
KLVERGISVQSYNGDLLYEPWEIYCEKGKPFTSFNSYWKKCLDMSIESVMLPPPWRLMP
ITAAAEAIWACSIEELGLENEAEKPSNALLTRAWSPGWSNADKLLNEFIEKQLIDYAKNS
KKVVGNSTSLLSPYLHFGEISVRHVFQCARMKQIIWARDKNSEGEESADLFLRGIGLRE
YSRYICFNFPFTHEQSLLSHLRFFPWDADVDKFKAWRQGRTGYPLVDAGMRELWATGW
MHNRIRVIVSSFAVKFLLLPWKWGMKYFWDTLLDADLECDILGWQYISGSIPDGHELD
RLDNPALQGAKYDPEGEYIRQWLPELARLPTEWIHHPWDAPLTVLKASGVELGTNYAK
PIVDIDTARELLAKAISRTREAQIMIGAAARDPPDLDNEFDSAGSAGSAGTSMVSKGEED
NMAIIKEFMRFKVHMEGSVNGHEFEIEGEGEGRPYEGTQTAKLKVTKGGPLPFAWDILS
PQFMYGSKAYVKHPADIPDYLKLSFPEGFKWERVMNFEDGGVVTVTQDSSLQDGEFIY
KVKLRGTNFPSDGPVMQKKTMGWEASSERMYPEDGALKGEIKQRLKLKDGGHYDAE
VKTTYKAKKPVQLPGAYNVNIKLDITSHNEDYTIVEQYERAEGRHSTGGMDELYKSGL
RSRVGGSGGSYYRKGGKALTANLWNWVNDACSSLSGNKESSGDRCAGSHSATSSQQE
VCEGILLMTREEKMVPEDGAGVCGPVCAAGGPWAEVRDSRTFTLVSEVETQGDLSRKI
PTEDEYTDRPSQPSTGSLLLIQQGSKSIPPFQEPLEVGENDSLSQCFTGTESTVDSEGCDF
TEPPSRTDSMPVSPEKHLTKEIEGDSCLPWVVSSNSTDGYTGSGNTPGEDHEPFPGSLKC
GPLPQCAYSMGFPSEAAASMAEAGVRPQDRADERGASGSGSSPSDQPPASGNVTGNSN
STFISSGQVMNFKGDIIVVYVSQTSQEGPGSAEPESEPVGRPVQEETLAHRDSFAGTAPR
FPDVCATGAGLQEQGAPRQKDGTSRPVQEQGGAQTSLHTQGSGQCAEGGSGGSGTGS
GATNFSLLKQAGDVEENPGPAPGSDSAGSAGMNGAIGGDLLLNFPDMSVLERQRAHLK
YLNPTFDSPLAGFFADSSMITGGEMDSYLSTAGLNLPMMYGETTVEGDSRLSISPETTLG
TGNFKAAKFDTETKDCNEAAKKMTMNRDDLVEEGEEEKSKITEQNNGSTKSIKKMKH
KAKKEENNFSNDSSKVTKELEKTDYIAPGSDSAGSAGMNGAIGGDLLLNFPDMSVLER
QRAHLKYLNPTFDSPLAGFFADSSMITGGEMDSYLSTAGLNLPMMYGETTVEGDSRLSI
SPETTLGTGNFKAAKFDTETKDCNEAAKKMTMNRDDLVEEGEEEKSKITEQNNGSTKS

IKKMKHKAKKEENNFSNDSSKVTKELEKTDYITGGGSSAGGSAGGSAGGKKKKKKSK
TKCVIM*

GFP-TRAF6

GFP
TRAF6 (mouse full coding sequence)

Nucleotide sequence

ATGGTGAGCAAGGGCGAGGAGCTGTTCACCGGGGTGGTGCCCATCCTGGTCGAGCT
GGACGGCGACGTAAACGGCCACAAGTTCAGCGTGTCCGGCGAGGGCGAGGGCGAT
GCCACCTACGGCAAGCTGACCCTGAAGTTCATCTGCACCACCGGCAAGCTGCCCGT
GCCCTGGCCCACCCTCGTGACCACCCTGACCTACGGCGTGCAGTGCTTCAGCCGCTA
CCCCGACCACATGAAGCAGCACGACTTCTTCAAGTCCGCCATGCCCGAAGGCTACGT
CCAGGAGCGCACCATCTTCTTCAAGGACGACGGCAACTACAAGACCCGCGCCGAGG
TGAAGTTCGAGGGCGACACCCTGGTGAACCGCATCGAGCTGAAGGGCATCGACTTC
AAGGAGGACGGCAACATCCTGGGGCACAAGCTGGAGTACAACTACAACAGCCACA
ACGTCTATATCATGGCCGACAAGCAGAAGAACGGCATCAAGGTGAACTTCAAGATCC
GCCACAACATCGAGGACGGCAGCGTGCAGCTCGCCGACCACTACCAGCAGAACACC
CCCATCGGCGACGGCCCCGTGCTGCTGCCCGACAACCACTACCTGAGCACCCAGTC
CGCCCTGAGCAAAGACCCCAACGAGAAGCGCGATCACATGGTCCTGCTGGAGTTCG
TGACCGCCGCCGGGATCACTCTCGGCATGGACGAGCTGTACAAGTCAGCAGGTGGT
AGTGCAGGAGGTGAATTCATGAGTCTCTTAAACTGTGAGAACAGCTGCGGGTCCAG
CCAGTCGTCCAGTGACTGCTGCGCTGCCATGGCCGCCTCCTGCAGCGCTGCAGTGA
AAGATGACAGCGTGAGTGGCTCTGCCAGCACCGGGAACCTCTCCAGCTCCTTCATG
GAGGAGATCCAGGGCTACGATGTGGAGTTTGACCCACCTCTGGAGAGCAAGTATGA
GTGTCCCATTTGCTTGATGGCTTTACGGGAAGCAGTGCAAACACCATGTGGCCACAG
GTTCTGCAAAGCCTGCATCATCAAATCCATAAGGGATGCAGGGCACAAGTGCCCAGT
TGACAATGAAATACTGCTGGAAAATCAACTGTTTCCCGACAATTTTGCAAAGCGAGA
GATTCTTTCCCTGACGGTAAAGTGCCCAAATAAAGGCTGTTTGCAAAAGATGGAACT
GAGACATCTCGAGGATCATCAAGTACATTGTGAATTTGCTCTAGTGAATTGTCCCCAG
TGCCAACGTCCTTTCCAGAAGTGCCAGGTTAATACACACATTATTGAGGATTGTCCCA
GGAGGCAGGTTTCTTGTGTAAACTGTGCTGTGTCCATGGCATATGAAGAGAAAGAGA
TCCATGATCAAAGCTGTCCTCTGGCAAATATCATCTGTGAATACTGTGGTACAATCCT
CATCAGAGAACAGATGCCTAATCATTATGATCTGGACTGCCCAACAGCTCCAATCCCT
TGCACATTCAGTGTTTTTGGCTGTCATGAAAAGATGCAGAGGAATCACTTGGCACGA

CACTTGCAAGAGAATACCCAGTTGCACATGAGACTGTTGGCCCAGGCTGTTCATAAT
GTTAACCTTGCTTTGCGTCCGTGCGATGCCGCCTCTCCATCCCGGGGATGTCGTCCAG
AGGACCCAAATTATGAGGAAACTATCAAACAGTTGGAGAGTCGCCTAGTAAGACAG
GACCATCAGATCCGGGAGCTGACTGCCAAAATGGAAACTCAGAGTATGTACGTGGG
CGAGCTCAAACGGACCATTCGGACCCTGGAGGACAAGGTTGCCGAAATGGAAGCAC
AGCAGTGTAACGGGATCTACATTTGGAAGATTGGCAACTTTGGGATGCACTTGAAAT
CCCAAGAAGAGGAAAGACCTGTTGTCATCCATAGCCCTGGATTCTACACAGGCAGA
CCTGGGTACAAGCTGTGCATGCGCCTGCATCTTCAGTTACCGACAGCTCAGCGCTGT
GCAAACTATATATCCCTTTTTGTCCACACAATGCAAGGAGAATATGACAGCCACCTCC
CCTGGCCCTTCCAGGGTACAATACGCCTTACAATTCTCGACCAGTCTGAAGCACTTAT
AAGGCAAAACCACGAAGAGGTCATGGACGCCAAACCAGAACTGCTTGCCTTTCAGC
GACCCACAATCCCACGGAACCCCAAAGGTTTTGGCTATGTAACATTTATGCACCTGG
AAGCCTTAAGACAGGGAACCTTCATTAAGGATGATACATTACTAGTGCGCTGTGAAG
TCTCTACCCGCTTTGACATGGGTGGCCTTCGGAAGGAGGGTTTCCAGCCACGAAGTA
CTGATGCGGGGGTGTAG

Amino acid sequence

MVSKGEELFTGVVPILVELDGDVNGHKFSVSGEGEGDATYGKLTLKFICTTGKLPVPWP
TLVTTLTYGVQCFSRYPDHMKQHDFFKSAMPEGYVQERTIFFKDDGNYKTRAEVKFEG
DTLVNRIELKGIDFKEDGNILGHKLEYNYNSHNVYIMADKQKNGIKVNFKIRHNIEDGS
VQLADHYQQNTPIGDGPVLLPDNHYLSTQSALSKDPNEKRDHMVLLEFVTAAGITLGM
DELYKSAGGSAGGEFMSLLNCENSCGSSQSSSDCCAAMAASCSAAVKDDSVSGSASTG
NLSSSFMEEIQGYDVEFDPPLESKYECPICLMALREAVQTPCGHRFCKACIIKSIRDAGHK
CPVDNEILLENQLFPDNFAKREILSLTVKCPNKGCLQKMELRHLEDHQVHCEFALVNCP
QCQRPFQKCQVNTHIIEDCPRRQVSCVNCAVSMAYEEKEIHDQSCPLANIICEYCGTILIR
EQMPNHYDLDCPTAPIPCTFSVFGCHEKMQRNHLARHLQENTQLHMRLLAQAVHNVN
LALRPCDAASPSRGCRPEDPNYEETIKQLESRLVRQDHQIRELTAKMETQSMYVGELKR
TIRTLEDKVAEMEAQQCNGIYIWKIGNFGMHLKSQEEERPVVIHSPGFYTGRPGYKLCM
RLHLQLPTAQRCANYISLFVHTMQGEYDSHLPWPFQGTIRLTILDQSEALIRQNHEEVM
DAKPELLAFQRPTIPRNPKGFGYVTFMHLEALRQGTFIKDDTLLVRCEVSTRFDMGGLR
KEGFQPRSTDAGV*

Supplementary Figure S1. Nucleotide and amino acid sequences for Opto-RANKc, Opto-RANKm, and GFP-TRAF6. Key sequences are highlighted.

TRAF6, tumor necrosis factor receptor-associated factor 6; RANK, receptor activator of nuclear factor-kB; GFP, green fluorescent protein

Supplementary Figure S2

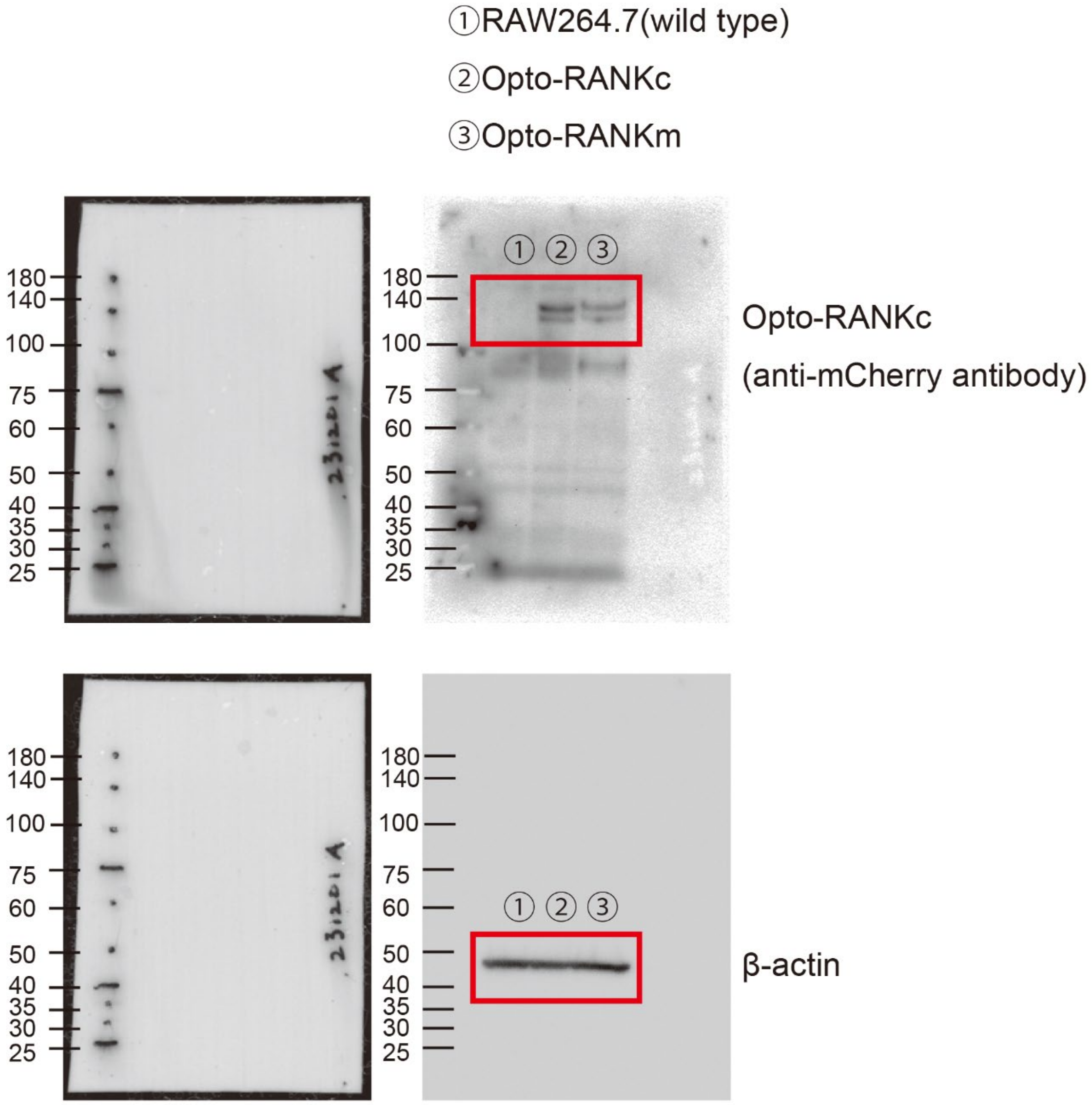

Supplementary Figure S2. Western blot detecting Opto-RANK proteins. The expression of Opto-RANKc and Opto-RANKm proteins in Opto-RANK cells was examined using a western blot with an anti-mCherry antibody. Both proteins were detected at approximately 130 kDa. Note that the CIBN-CIBN-CAAX at the C-terminus of Opto-RANKm is separated due to the P2A peptide, resulting in the detection of Opto-RANKc and Opto-RANKm at similar sizes. The protein extract from RAW264.7 cells was used as a negative control, and β-actin (approximately 42 kDa) was used as a loading control.

Supplementary Figure S3

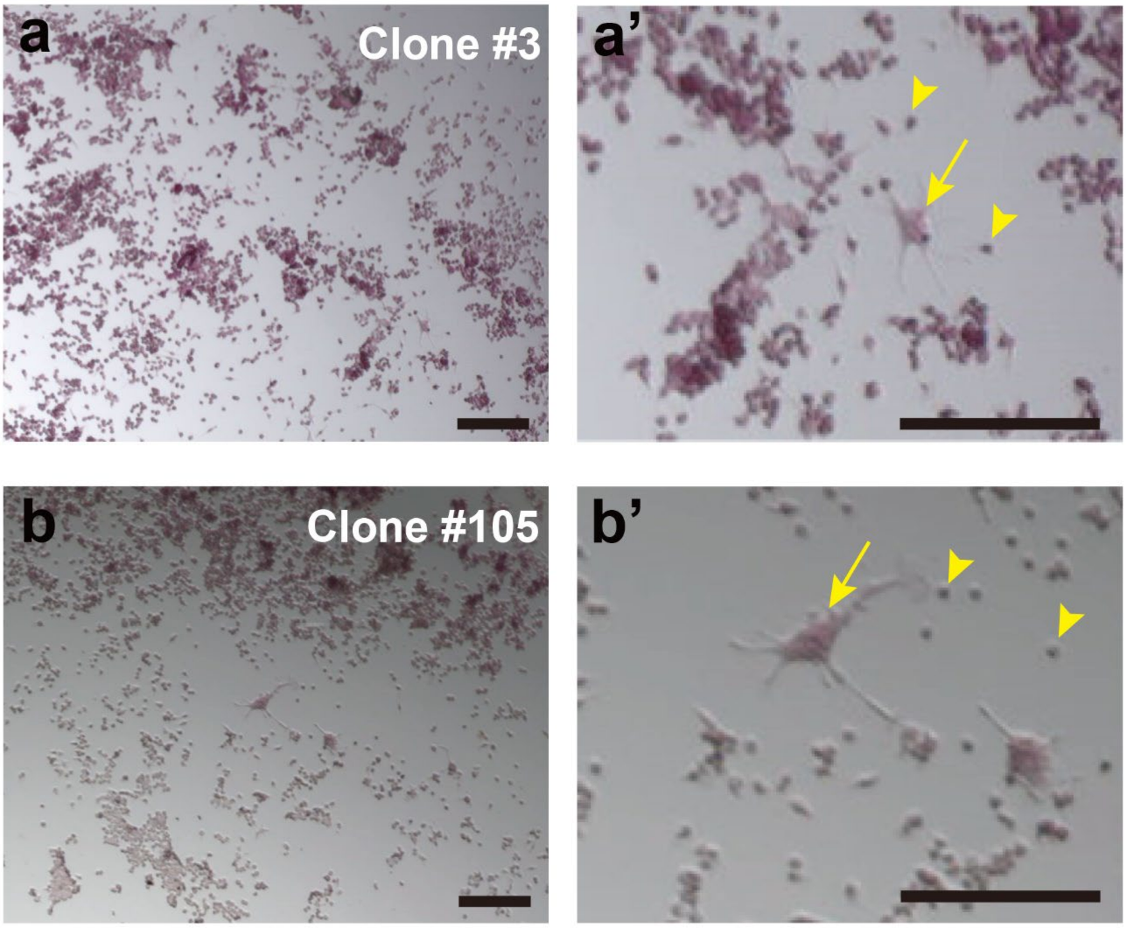

Supplementary Figure S3. Differentiation of Opto-RANKc cells from different clonal cell lines upon blue light illumination. Opto-RANKc cells (clone nos. 3 and 105), different from clone no. 110, which was intensively analyzed in this study, were cultured on collagen-coated plates with blue light illumination for 5 d. The cells were stained for TRAP. Most cells were TRAP-positive, and multinucleated cells were also observed. (a) Opto-RANKc cells (clone no. 3). (a′) A higher magnification view of the staining in (a). (b) Opto-RANKc cells (clone no. 105). (b′) A higher magnification view of the staining in (b). Arrows and arrow heads indicate multinucleated cells and mononuclear cells, respectively. Scale bar: 150 μm.

RANK, receptor activator of nuclear factor-kB; TRAP, tartrate-resistant acid phosphatase

Supplementary Figure S4

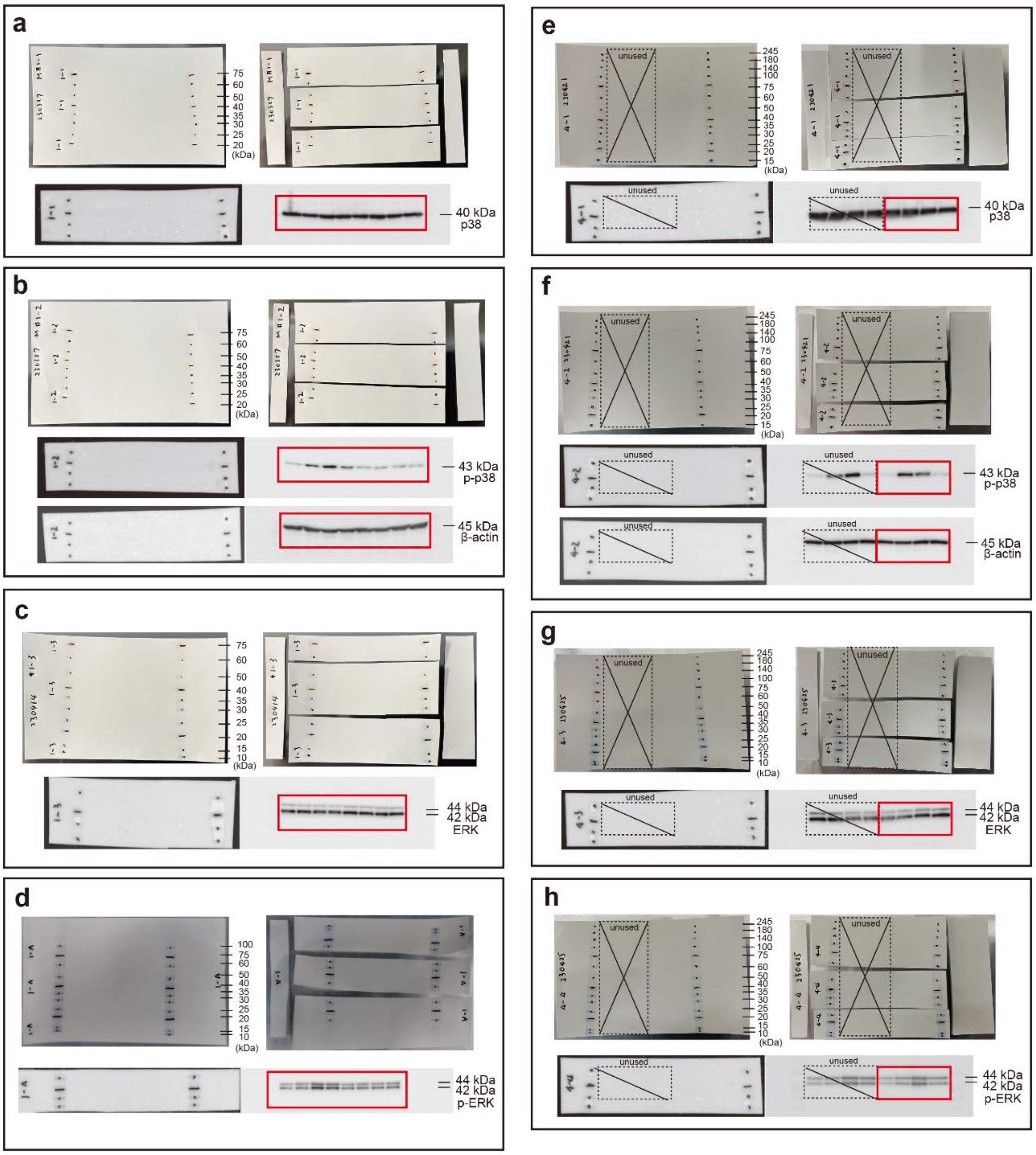

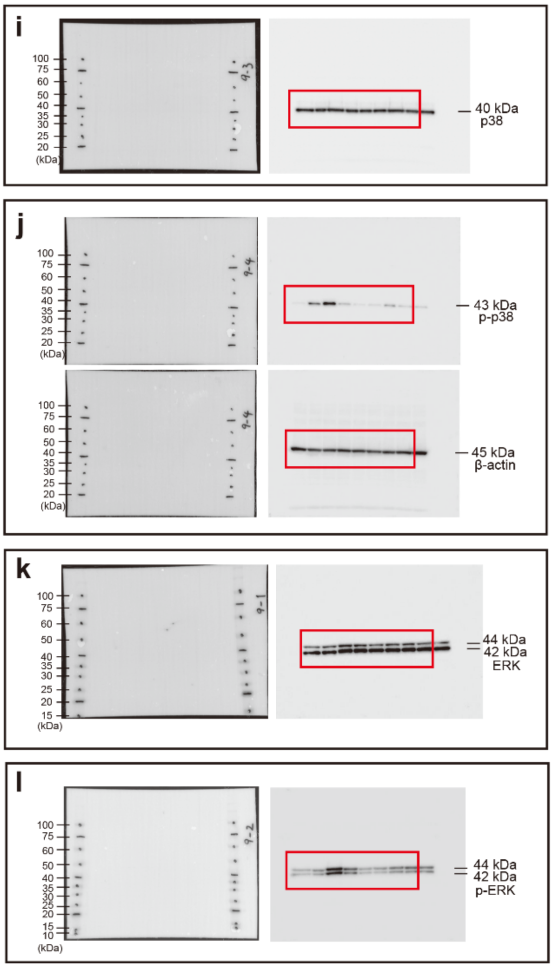

Supplementary Figure S4. Full-size membrane images of the western blots in Fig. 3. (a) p38 in Fig. 3a. (b) p-p38 and β-actin in Fig. 3a. (c) ERK in Fig. 3a. (d) p-ERK in Fig. 3a. (e) p38 in Fig. 3d (left). (f) p-p38 and β-actin in Fig. 3d (left). (g) ERK in Fig. 3d (left). (h) p-ERK in Fig. 3d (left). (i) p38 in Fig. 3d (middle and right). (j) p-p38 and β-actin in Fig. 3d (middle and right). (k) ERK in Fig. 3d (middle and right). (l) p-ERK in Fig. 3d (middle and right).

ERK, extracellular signal-regulated kinase; p-ERK, phosphorylated ERK

Supplementary Figure S5

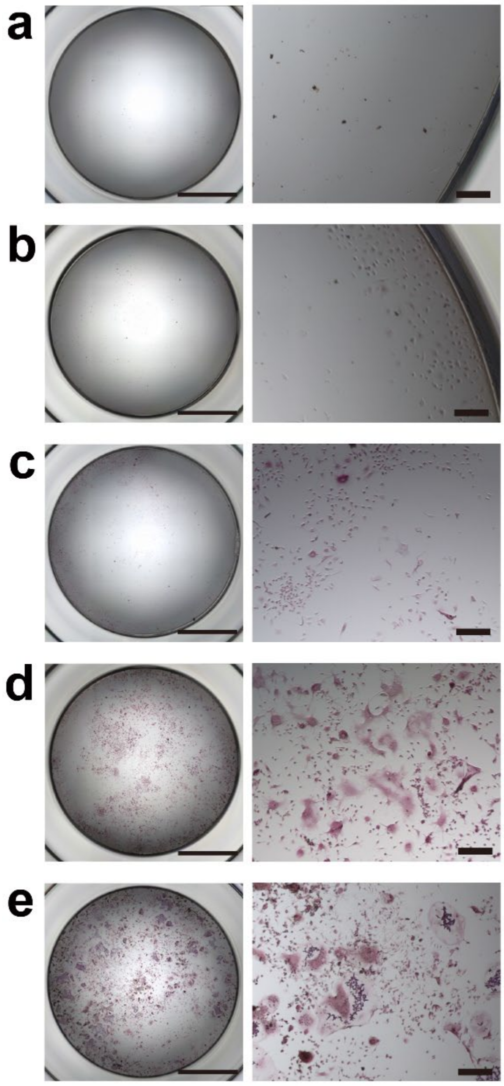

Supplementary Figure S5. Effect of various conditions of blue light illumination on RAW264.7 cell differentiation. Cells were cultured in a medium containing RANKL for 5 d, with various frequencies of blue light illumination. The cells were stained for TRAP, and representative images from nine independent experiments were shown. Note that dead cells and cells without differentiation were easily detached and disappeared during TRAP staining. Each light exposure below consists of 12 cycles of 10 ms irradiation, with a 100-ms interval. Blue light (wavelength=470 nm) exposure was carried out at the rate of every (a) 10 s, (b) 30 s, (c) 1 min, and (d) 2 min. (e) No blue light exposure. Scale bar: 2 mm (left) and 150 µm (right). The numbers of osteoclasts (>1500 μm^2) per well in 96-well plates in (d) and (e) are 160.3 ± 18.7 and 230.7 ± 59.8, respectively (mean ± SD from three independent experiments).

RANKL, receptor activator of nuclear factor-kB ligand; TRAP, tartrate-resistant acid phosphatase

Supplementary Figure S6

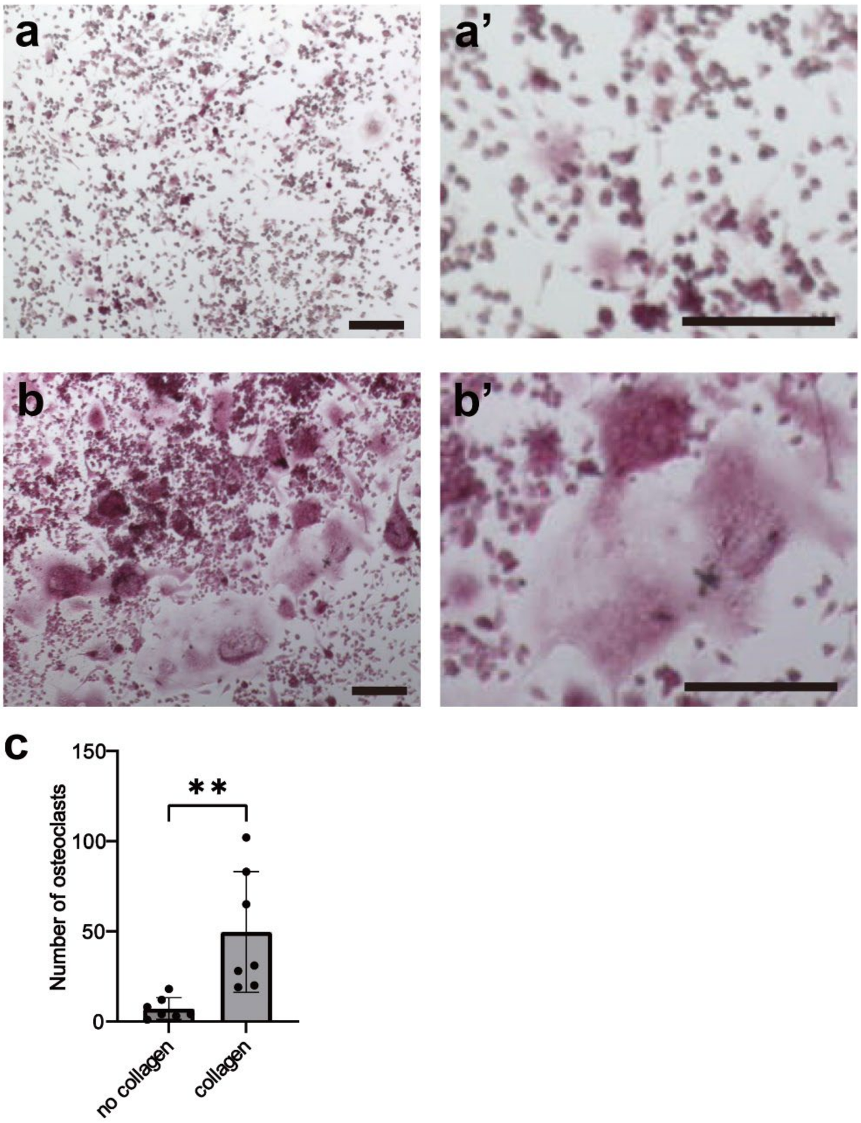

Supplementary Figure S6. Effect of collagen coating of culture dishes on osteoclast differentiation. Opto-RANKc cells were cultured on plates with/without collagen coating, with blue light illumination for 7 d; the cells were passaged after 4 d of culture. The cells were then stained for TRAP. Representative images are shown (*n*=7 cultures from three independent experiments). (a) Cells cultured on a non-coated plate. (a′) A higher magnification view of the staining in (a). (b) Cells cultured on a collagen-coated plate. (b′) A higher magnification view of the staining in (b). (c) The number of multinucleated cells (size >1500 μm^2) per well. Data were analyzed using a two-tailed unpaired *t*-test. **, p<0.01. Scale bar in (a)–(b′): 150 µm.

RANK, receptor activator of nuclear factor-kB; TRAP, tartrate-resistant acid phosphatase

Supplementary Figure S7

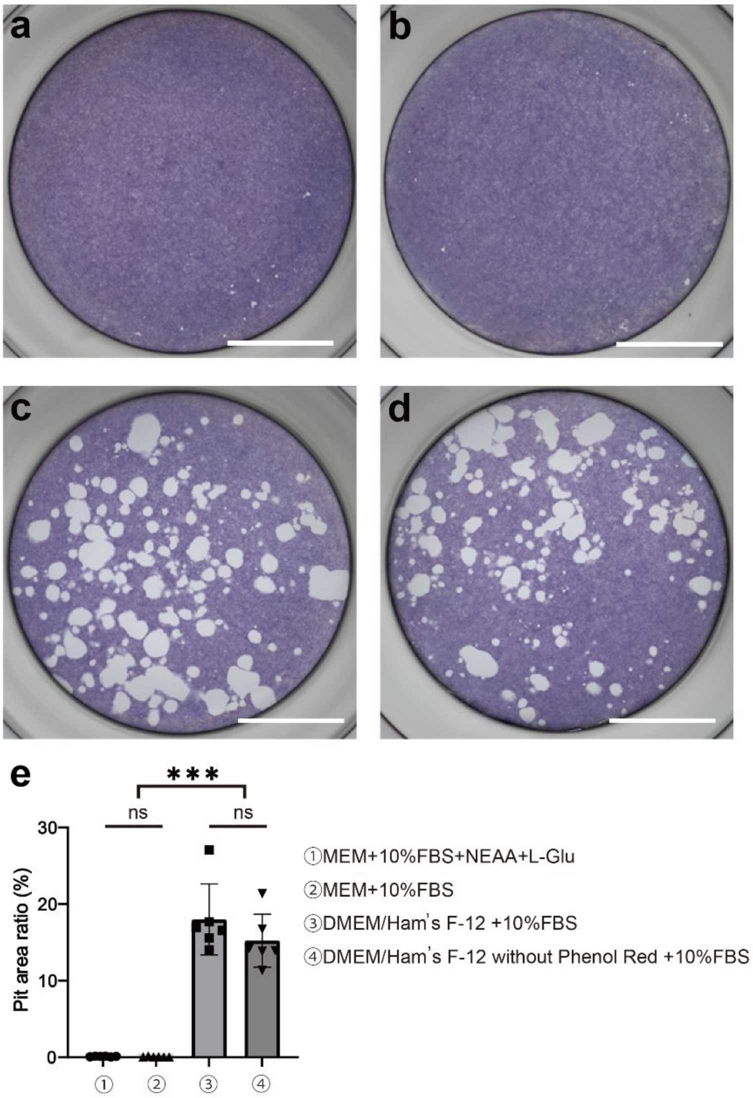

Supplementary Figure S7. Osteoclast pit-formation in various media. RAW264.7 cells were cultured in various media containing RANKL on CaP-coated plates for 7 d. Representative images of pit-formation upon use of (a) MEM containing 10% FBS supplemented with NEAA mixture and L-GLU, (b) MEM containing 10% FBS, (c) DMEM/Ham's F-12 (with Phenol Red) containing 10% FBS, and (d) DMEM/Ham's F-12 (without Phenol Red) containing 10% FBS. (e) The percentage of the pit area to the total well area, represented as mean ± SD from six independent experiments. Data were analyzed using a one-way analysis of variance followed by Tukey's multiple-comparisons test. ***p<0.001. Scale bar in (a)–(d): 2 mm.

CaP, calcium phosphate; MEM, Minimal Essential Medium; FBS, fetal bovine serum; NEAA, non-essential amino acid; L-GLU, L-glutamine

Supplementary Figure S8

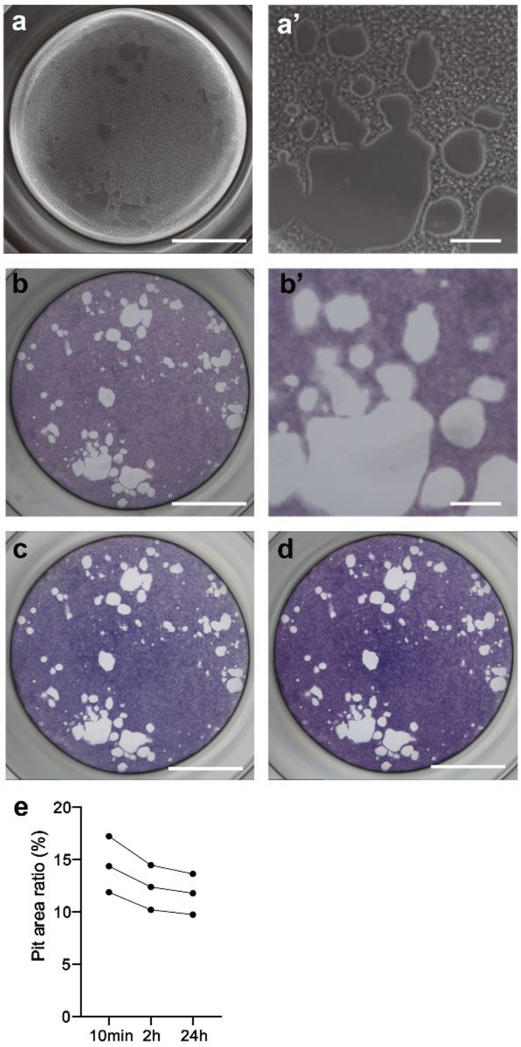

Supplementary Figure S8. **Trypan Blue dye has no effect on CaP coatings.** Prolonged staining of CaP coating with Trypan Blue dye results in a darker blue color, which makes the pit apparently smaller in automated measurements. (a) A CaP-coated plate with pits formed by differentiated RAW264.7 cells. Scale bar: 2 mm. (a′) A high magnification view of (a). Scale bar: 300 µm. (b) The CaP-coated plate with Trypan Blue staining for 10 min. Scale bar: 2 mm. (b′) A high magnification view of (b). Scale bar: 300 µm. (c) The CaP-coated plate with Trypan Blue staining for a further 2 h. Scale bar: 2 mm. (d) The CaP-coated plate with Trypan Blue staining for a further 24 h. Scale bar: 2 mm. (e) The percentage of the pit area to that of the entire well area in (b–d), represented as mean ± SD from three independent experiments. Data were analyzed using a two-tailed paired *t*-test. $p=0.023$ (upon comparing the 10-min and 2-h data); $p=0.029$ (upon comparing the 2-h and 24-h data).

CaP, calcium phosphate
